# Slow-fast analysis of a multi-group asset flow model with implications for the dynamics of wealth

**DOI:** 10.1371/journal.pone.0207764

**Published:** 2018-11-29

**Authors:** Mark DeSantis, David Swigon

**Affiliations:** 1 Argyros School of Business and Economics, Chapman University, Orange, CA, United States of America; 2 Department of Mathematics, University of Pittsburgh, Pittsburgh, PA, United States of America; Central South University, CHINA

## Abstract

The multi-group asset flow model is a nonlinear dynamical system originally developed as a tool for understanding the behavioral foundations of market phenomena such as flash crashes and price bubbles. In this paper we use a modification of this model to analyze the dynamics of a single-asset market in situations when the trading rates of investors (i.e., their desire to exchange stock for cash) are prescribed ahead of time and independent of the state of the market. Under the assumption of fast trading compared to the time-rate of change in the prescribed trading rates we decompose the dynamics of the system to fast and slow components. We use the model to derive a variety of observations regarding the dynamics of price and investors’ wealth, and the dependence of these quantities on the prescribed trading rates. In particular, we show that strategies with constant trading rates, which represent the well-known constant-rebalanced portfolio (CRP) strategies, are optimal in the sense that they minimize investment risks. In contrast, we show that investors pursuing non-CRP strategies are at risk of loss of wealth, as a result of the slow system not being integrable in the sense that cyclic trading rates do not always result in periodic price variations.

## Introduction

Modeling of price and wealth dynamics is traditionally based upon the efficient market hypothesis, which in its semi-strong form suggests that all publicly available information is already reflected in asset prices. Thus, any pricing mistakes caused by behavioral biases or cognitive errors are immediately exploited by rational investors with ample (usually assumed to be infinite) capital [1]. As such, these mispricings are not systematic and asset prices may be modeled as random processes [2]. This approach yields many valuable insights and forms the foundation for modern studies of price dynamics and option pricing. However, there are many phenomena, such as price bubbles and “flash crashes” that have significant economic repercussions, and yet are not explained by this classical model. This has led to the existence and growth of the field of Behavioral Finance (see, e.g. [3]) and the development of a variety of new models (e.g., [4–6]) designed to deal with situations in which investors do not act rationally, i.e., they do not immediately (and correctly) update their belief of the true value of the asset upon receiving new information and make decisions that maximize their utility [7]. These models account for factors such as the recent trend in price and behavioral biases such as conservatism (individuals are slow to update their beliefs when presented with new evidence), the representativeness heuristic (individuals assess the probability of an event based upon its similarity to an event with which the individuals are familiar), and overconfidence.

One such model, named the *multi-group asset flow model* uses differential equations to study the dynamics of asset prices and investor wealth without making assumptions regarding an infinite supply of capital, shares, and/or traders, or any external assumptions about price evolution and volatility [8–14]. It has proven effective in describing the evolution of asset prices in both experimental asset markets [15] and real world financial markets [10]. The model has also provided insights into the bubble-bust phenomenon [13] by showing how a change in the timeframe of interest for trend-based traders can result in a “flash crash,” where the price drops within a short time by a significant amount. Originally, this model focused on a single group of traders who focused not only on the intrinsic value of the asset, but also the recent trend in price [8, 16]. The model was extended in [11] to accommodate multiple investor groups and then used to offer insights into the stability of prices [17, 18]. Indeed, the model admits a range of both stable and unstable equilibria. Thus prices may start near an unstable equilibrium and eventually settle near a stable equilibrium that may be very close to the initial price. However, the price path (or excursion) from one equilibria to another may be quite large and therefore economically significant [18]. None of the previous studies on the multi-group asset flow model performed a slow-fast analysis of the dynamics of the system nor treated traders’ strategies as predetermined inputs to the system. The model behavior is similar to those of other studies that have considered heterogeneity in investor beliefs within deterministic price models and attempted to explain market phenomena as consequences of the (deterministic) trading behaviors of heterogeneous agents [10, 19–22].

As described in [11] and [17], the pricing equation in the multi-group asset flow model is based on the standard microeconomic principle that the return is proportional to excess demand (see [23]). Indeed, we start with the following price equation

$$\begin{array}{c} \tau \frac{1}{P}\frac{dP}{dt}=\frac{D-S}{S} \end{array}$$ (1)

where *τ* is a time scale characterizing the speed of the market. In the formulation of [17] and [18], the multi-group asset flow model has the form:

$$\begin{array}{c} \tau \frac{dP}{dt}=F-P \end{array}$$ (2)

$$\begin{array}{c} \tau \frac{dN_{i}}{dt}=\frac{k_{i}M_{i}}{F}-{\tilde{k}}_{i}N_{i}\;i=1,2,\ldots ,G \end{array}$$ (3)

$$\begin{array}{c} \tau \frac{dM_{i}}{dt}=-k_{i}M_{i}+{\tilde{k}}_{i}N_{i}F\;i=1,2,\ldots ,G \end{array}$$ (4)

and describes *G* investors (or investor groups), each endowed with two types of assets (shares of a single non-dividend paying stock in the amount *N*_*i*_ and cash in the amount *M*_*i*_), who exchange these assets at rates specific to each investor and at a price *P* that is determined by the ratio *F* of demand and supply:

$$\begin{array}{c} F=\frac{\sum_{i=1}^{G}k_{i}M_{i}}{\sum_{i=1}^{G}{\tilde{k}}_{i}N_{i}}. \end{array}$$ (5)

The investor trading preferences are characterized by the trading rate functions, *k*_*i*_(*t*) and ${\tilde{k}}_{i}\left(t\right)$, which are to be interpreted as follows: The quantity *k*_*i*_*M*_*i*_ represents the rate at which investor *i* uses cash to buy stock, i.e., the proportion of cash that investor *i* submits per unit of time to purchase shares. Similarly, the quantity ${\tilde{k}}_{i}N_{i}$ represents the rate at which investor *i* sells the stock, i.e., the proportion of shares that investor *i* submits for sale per unit of time. Hence, the total supply (in terms of dollars), *SP*, of shares for sale in the market corresponds to $\sum_{i=1}^{G}{\tilde{k}}_{i}N_{i}P$, while the demand (in terms of dollars), *D*, is represented as $\sum_{i=1}^{G}k_{i}M_{i}$. Thus, (2) is obtained from (1) by substituting the appropriate values for *S* and *D*. (We direct the interested reader to [17] for a detailed description of the model’s derivation as a limiting case of discrete-time trading assumptions.)

The dynamics of the system (2)–(5) is determined by the market participants’ trading rates, *k*_*i*_ and ${\tilde{k}}_{i}$, which vary in time according to the investors’ strategies. In the original design of the multi-group asset model, the functions *k*_*i*_(*t*) and ${\tilde{k}}_{i}$ were chosen to be functionals of the history of *P* or *dP*/*dt* up to the time *t*, reflecting situations in which traders make decisions to buy/sell based upon the recent price’s deviation from some fundamental value (fundamental or value investors) or the recent direction and magnitude of price changes (momentum or trend-based investors) [17, 18], a choice that is traditionally made in models of behavioral finance. In contrast, in this paper we focus on a different situation in which each trader in the market specifies the trading rate as a prescribed function of time, *k*_*i*_(*t*), independent of the dynamics of the market. Thus, the investor’s motivation to buy/sell is not reactive but *pre-planned*. For example, the investor may choose to follow the so called *constant rebalanced portfolio (CRP) strategy* in which the proportion of investor wealth in different assets remains constant over a period of time by setting *k*_*i*_(*t*) = *const*. The investor then effectively rebalances his portfolio by selling assets after they increase in price and buying after they decrease in price [24–27]. (CRP is also referred to as a “constant-mix” or “fixed-mix” asset allocation strategy. A special case is the 1/*n* investment allocation, which directs an individual holding a portfolio containing *n* assets to maintain a 1/*n* proportion of wealth invested in each asset [28].) Another example of a pre-planned strategy is that of an investor with a long-term investment horizon who maintains a percentage of 100 minus his age in stocks and the remainder in bonds, i.e., *k*_*i*_(*t*) = 1 − *αt*; this strategy is common for retirement accounts, e.g., StateFarm LifePath funds.

The objective of this paper is to evaluate the performance of the CRP strategy and other investment strategies, expressed as functions *k*_*i*_(*t*), by analyzing their effect on the price and investors’ wealth. Since the behavior of the market depends on the strategies of all investors participating in it, a change in *k*_*i*_(*t*) for one investor leads to changes in the wealth of all investors. We provide evidence supporting the use of CRP trading strategy as a default approach in the dynamical system model if the strategies of other investors are not known in advance or if the potential for loss of wealth is to be minimized. If others’ strategies are known, then we offer improvements to this baseline heuristic and define strategies that lead to gains in wealth. Most of the results we present can be considered intuitive. For example, it is known that if an investor knows in advance the strategy (plan of future market orders) of another trader, then the investor can take advantage of this information and increase his wealth at the expense of the other trader by preemptively performing the same sequence of market orders. This is similar to the predatory trading strategy described in [29] and [30]. Our results confirm this intuitive conclusion by providing mathematical validation within the constructs of the model, and by quantifying the magnitude of the wealth gain or loss as a function of the strategies chosen by the investors.

In our analysis we focus primarily on cyclic strategies in which *k*_*i*_(*t*) are periodic functions of time with the same period, i.e., strategies in which the proportions of investors’ wealth invested in the asset return to their starting values. We do this to better compare the relative gain or loss in wealth of the investors by eliminating the gains from an overall increase in demand for the asset (or losses from an overall decrease in demand). In this sense, our paper analyzes trading gains and losses as opposed to market gains and losses, in the spirit of [31]. For simplicity, we assume that the market equilibrates on a time-scale *τ* that is shorter than the time-scale of changes in trading rates of the investors, which reflects today’s trading speed of electronic markets. This assumption leads to the separation of the dynamics of the system (2)–(5) into fast and slow regimes.

The paper is organized as follows: the model framework and the dynamical system describing the market are introduced in the following section. The reduction of the system to the slow time-scale and the resulting quasi-steady market dynamics formulation is stated in the “Slow-fast analysis” section. The main findings are derived in the section “Effects of strategies on investor wealth” and subsequently illustrated in examples presented in the “Numerical results” section.

## Model setup

As stated above, in this paper we study the multi-group asset flow model (2)–(5). The price *P*, amounts of stock *N*_*i*_ and amounts of cash *M*_*i*_ are assumed to be non-negative, i.e., we do not allow short selling of shares or borrowing of cash. The initial conditions are defined as *P*(0) = *P*_0_, *N*_*i*_(0) = *N*_*i*,0_, and *M*_*i*_(0) = *M*_*i*,0_, with *i* = 1, 2, …, *G*. The system (2)-(6) has two conserved quantities, namely the total amount of cash $\sum_{i=1}^{G}M_{i}=\bar{M}$ and the total number of shares $\sum_{i=1}^{G}N_{i}=\bar{N}$ and hence represents trading within a closed system [18].

In contrast with [18], the trading rates *k*_*i*_ and ${\tilde{k}}_{i}$ are here treated as inputs or controls for the system. For simplicity, just as in [17] and [18], we assume that *k*_*i*_(*t*) ≥ 0, ${\tilde{k}}_{i}\left(t\right)\geq 0$, and

$$\begin{array}{c} k_{i}\left(t\right)+{\tilde{k}}_{i}\left(t\right)=1. \end{array}$$ (6)

i.e., each is both buying and selling the asset continuously as long as 0 < *k*_*i*_(*t*) < 1. As observed by [18], this assumption has an important implication: at constant *k*_*i*_, *F* in (5) is constant and (2)–(4) reduce to a system of linear equations (see also the “Slow-fast analysis” section below). We do not wish to elaborate on the mechanism by which trading is to be realized in practice, but one possibility is that the investor continuously maintains unfulfilled buy or sell orders in the market. Note that *k*_*i*_(*t*) = 1 corresponds to the (all-in) “buy and hold” strategy, where the investor exchanges all of his cash for shares, while *k*_*i*_(*t*) = 0 corresponds to the investor divesting himself of all shares (i.e., exiting the market).

The wealth of an investor can be measured by the total cash value of all assets the investor owns:

$$\begin{array}{c} W_{i}=M_{i}+N_{i}P \end{array}$$ (7)

In view of (2)–(4), the time-rate of change of an individual investor’s wealth is given by

$$\begin{array}{ll} \frac{dW_{i}}{dt} & =N_{i}\frac{dP}{dt}+\frac{dN_{i}}{dt}P+\frac{dM_{i}}{dt}\\ & =N_{i}\frac{dP}{dt}+\frac{dN_{i}}{dt}\left(P-F\right)\\ & =N_{i}\frac{dP}{dt}+\frac{dN_{i}}{dt}\left(-\tau \frac{dP}{dt}\right)\\ & =\left(N_{i}-\tau \frac{dN_{i}}{dt}\right)\frac{dP}{dt}, \end{array}$$ (8)

which implies (not surprisingly) that when the price is constant, there is no change in the wealth of any investor. It also implies that instantaneous positive growth of wealth can be achieved by gradual buying, holding, or selling of the asset when the price is increasing (with *dN*_*i*_/*dt* below *N*_*i*_/*τ*) and buying of the asset when the price is decreasing (i.e., by keeping *dN*_*i*_/*dt* above *N*_*i*_/*τ*). Of course, any action taken by the investor will result in a market reaction that will influence the future price.

The dynamics of wealth, *W*_*i*_, can be better understood by analyzing a dynamical system equivalent to (2)–(6) in which cash is replaced with wealth as a dependent variable:

$$\begin{array}{c} \tau \frac{dP}{dt}=F-P \end{array}$$ (9)

$$\begin{array}{c} \tau \frac{dN_{i}}{dt}=\frac{k_{i}W_{i}}{F}-(1-k_{i}\frac{F-P}{F})N_{i} \end{array}$$ (10)

$$\begin{array}{c} \tau \frac{dW_{i}}{dt}=[-k_{i}W_{i}+(2-k_{i}\frac{F-P}{F})N_{i}F]\frac{F-P}{F} \end{array}$$ (11)

with *i* = 1, 2, …, *G*, where

$$\begin{array}{c} F=\frac{\sum_{i=1}^{G}k_{i}W_{i}-P\sum_{i=1}^{G}k_{i}N_{i}}{\sum_{i=1}^{G}\left(1-k_{i}\right)N_{i}} \end{array}$$ (12)

Note that Eqs (9)–(12) represent a closed system of differential equations with solution depending uniquely on the initial conditions *P*(0) = *P*_0_, *N*_*i*_(0) = *N*_*i*,0_, *W*_*i*_(0) = *W*_*i*,0_, *i* = 1, …, *G*, and the input functions *k*_*i*_(*t*).

The *constant rebalanced portfolio* (CRP) strategy can be naturally represented within the model (2)–(6) (or, equivalently, the system (9)–(12)) as a strategy with a constant trading rate *k*_*i*_. This is justified by the following considerations: By adopting a fixed *k*_*i*_ strategy, the investor has a guarantee that whenever the system reaches equilibrium, the proportion of his wealth in the stock, *N*_*i*_ *P*/*W*_*i*_, will be equal to *k*_*i*_ and the proportion of his wealth in cash, *M*_*i*_/*W*_*i*_, will be 1 − *k*_*i*_ (see also [18]). Outside of equilibrium these proportions will not necessarily be maintained due to a delay in the approach to equilibrium. However, the system will always move in the direction of the appropriate wealth proportion defined by *k*_*i*_, i.e., the signs of *dM*_*i*_/*dt* and *dN*_*i*_/*dt* will be such that the absolute difference |*k*_*i*_ *M*_*i*_ − (1 − *k*_*i*_)*N*_*i*_ *P*| decreases. In the context of the model (2)–(6) the 1/*n* strategy is represented by *k*_*i*_ = 1/2, since there are only two assets in each investor’s portfolio: cash and stock.

In the subsequent section we shall occasionally make use of vector notation with **k** = [*k*_1_, *k*_2_, …, *k*_*G*_], **M** = [*M*_1_, *M*_2_, …, *M*_*G*_], **N** = [*N*_1_, *N*_2_, …, *N*_*G*_], and **W** = [*W*_1_, *W*_2_, …, *W*_*G*_]. In that notation, $\sum_{i=1}^{G}k_{i}W_{i}$ can be written as the dot product **k** ⋅ **W**, while $\sum_{i=1}^{G}\left(1-k_{i}\right)N_{i}$ can be written as (**1** − **k**) ⋅ **N** where **1** = [1, 1, …, 1], etc.

## Slow-fast analysis

In this and the remaining sections of the paper we will treat *τ* as a small parameter and study the dynamics of the system (2)–(5) in the limit as *τ* approaches zero, which is the case, for example, for fast equilibrating markets that allow high-frequency trading. In traditional slow-fast systems, the smallness parameter shows up in equations for a subset of the variables of the system, and the system exhibits two distinct dynamical regimes: (i) rapid approach to slow manifold and (ii) gradual movement along the slow manifold.

In the present case, the slow variables are the *t*-dependent functions *k*_*i*_(*t*). Since in the Eqs (2)–(6) the time rate of change of every variable of the system is multiplied by *τ*, the *fast dynamics* of that system can be characterized as a rapid approach to an equilibrium when all trading rates *k*_*i*_ are constant. For constant *k*_*i*_, (2)–(6) reduces to a system of linear ODEs with *G* − 1-dimensional equilibrium manifold [18]

$$\begin{array}{c} E_{M}=\{\left(P,N,M\right)|N\cdot 1=\bar{N},\;M\cdot 1=\bar{M},\;\frac{k_{i}M_{i}}{\left(1-k_{i}\right)N_{i}P}=1,i=1,\ldots ,G\} \end{array}$$ (13)

From any initial condition (*P*_0_, **N**_0_, **M**_0_) the system approaches $E_{M}$ along a linear trajectory given by

$$\begin{array}{c} P(t)=P^{*}+\left(P_{0}-P^{*}\right)e^{-\tau t} \end{array}$$ (14)

$$N\left(t\right)=N^{*}+\left(N_{0}-N^{*}\right)e^{-\tau t}$$ (15)

$$M\left(t\right)=M^{*}+\left(M_{0}-M^{*}\right)e^{-\tau t}$$ (16)

where the equilibrium state $\left(P^{*},N^{*},M^{*}\right)\in E_{M}$ obeys P* = (**k** ⋅ **M**_0_)/((**1** − **k**) ⋅ **N**_0_), $N_{i}^{*}=k_{i}\left(M_{i,0}/P^{*}+N_{i,0}\right)$, and $M_{i}^{*}=\left(1-k_{i}\right)\left(M_{i,0}+N_{i,0}P^{*}\right)$ [18].

Analogously, the system (9)–(12) converges to the *G* − 1 dimensional manifold

$$\begin{array}{c} E_{W}=\{\left(P,N,W\right)|N\cdot 1=\bar{N},\;W\cdot 1=\bar{M}+\bar{N}P,\;\frac{k_{i}W_{i}}{N_{i}P}=1,i=1,\ldots ,G\} \end{array}$$ (17)

with *P*(*t*) and **N**(*t*) given by (14) and (15), and with

$$\begin{array}{c} W\left(t\right)=W^{*}+\left(W_{0}-W^{*}\right)e^{-2\tau t}+N^{*}\left(P_{0}-P^{*}\right)\left(1-e^{-\tau t}\right)e^{-\tau t} \end{array}$$ (18)

where W* = **M**_0_ + **N**_0_ P*. (Note that the last term in the above equation vanishes both at *t* = 0 and as *t* → ∞.)

The *slow dynamics of the system* is due to the time-dependence of the trading rates *k*_*i*_ which results in a time-dependence of the equilibrium manifold. We can derive the reduced equations for such dynamics (also called the slow subsystem) by equating the lowest order terms in *τ* in each of the Eqs (9)–(11). The order of terms in those equations can be better ascertained by rewriting the equations in the following way:

$$\begin{array}{c} \tau \frac{dP}{dt}=F-P \end{array}$$ (19)

$$\begin{array}{c} \tau \frac{dN_{i}}{dt}=\frac{k_{i}W_{i}}{F}-(1-\tau \frac{k_{i}}{F}\frac{dP}{dt})N_{i} \end{array}$$ (20)

$$\begin{array}{c} \frac{dW_{i}}{dt}=-\tau \frac{dN_{i}}{dt}\frac{dP}{dt}+N_{i}\frac{dP}{dt} \end{array}$$ (21)

with *i* = 1, 2, …, *G*. By taking the limit *τ* → 0, we obtain the slow subsystem

$$\begin{array}{c} 0=F-P \end{array}$$ (22)

$$\begin{array}{c} 0=\frac{k_{i}W_{i}}{F}-N_{i} \end{array}$$ (23)

$$\begin{array}{c} \frac{dW_{i}}{dt}=N_{i}\frac{dP}{dt} \end{array}$$ (24)

with *i* = 1, 2, …, *G*. Note that, in view of the Eqs (22) and (23), the expression for *F* in (12) becomes an identity in the slow subsystem. The system is completed by providing an equation for *dP*/*dt* which can be obtained by differentiation of (12) while assuming that the functions *k*_*i*_(*t*) are bounded away from 0 and 1, continuously differentiable for all *t*. (See S1 Text.) In summary, the slow subsystem is given by:

$$\begin{array}{c} \frac{dP}{dt}=\frac{P\sum_{i=1}^{G}\frac{dk_{i}}{dt}W_{i}}{\sum_{i=1}^{G}\left(1-k_{i}\right)k_{i}W_{i}} \end{array}$$ (25)

$$\begin{array}{c} \frac{dW_{i}}{dt}=\frac{k_{i}W_{i}}{P}\frac{dP}{dt},\;i=1,\cdots ,G \end{array}$$ (26)

with initial conditions *P*(0) = *P*_0_, **W**(0) = **W**_0_. For any solution (*P*(*t*), **W**(*t*)) we can recover **M**(*t*) and **N**(*t*) using Eqs (23) and (22) as *N*_*i*_(*t*) = *k*_*i*_(*t*)*W*_*i*_(*t*)/*P*(*t*) and *M*_*i*_(*t*) = (1 − *k*_*i*_(*t*))*W*_*i*_(*t*). The conservation laws for shares and cash imply that any solution of (25)-(26) obeys the relations

$$\begin{array}{c} \sum_{i=1}^{G}k_{i}\left(t\right)W_{i}\left(t\right)=\bar{N}P\left(t\right),\;\sum_{i=1}^{G}\left(1-k_{i}\left(t\right)\right)W_{i}\left(t\right)=\bar{M} \end{array}$$ (27)

Note that the solution of (25)-(26) lies on the equilibrium manifold $E_{W}$. Since we are solving the dynamics of a quasi-steady state process, we must take the initial values to be in $E_{W}$ as well. Any initial values *P*_0_ and **W**_0_ for which (27) are satisfied at *t* = 0 describe such an equilibrium. (The constants $\bar{M}$ and $\bar{N}$ can be set in advance or determined by the initial conditions via (27).)

The following lemma describes some general observations about the dependence of the solutions of the system (25)-(26) on the trading rates *k*_*i*_(*t*), which are essential for the proofs of all remaining results in this paper.

**Lemma 1**. *Let (P(t)*, **W**(*t*)) *be a solution of the system* (25)-(26) *on the interval* [0, *T*] *with trading rates* **k**(*t*) *and initial conditions P*(0) = *P*_0_, **W**(0) = **W**_0_. *It follows that*

1. *d***k**(*t*)/*dt* ⋅ **W**(*t*) >(<) 0 *for t* ∈ [0, *T*] *if and only if dP*(*t*)/*dt* >(<) 0 *and*, *consequently*, *dW*_*i*_(*t*)/*dt* >(<) 0 *for t* ∈ [0, *T*] *for all i*, 1 ≤ *i* ≤ *G*.
2. *If* (**k**(*t*) − **k**(0)) ⋅ **W**_0_ = 0 *for t* ∈ [0, *T*], *then* **W**(*t*) = **W**_0_ *for t* ∈ [0, *T*].
3. *If dk*_*i*_(*t*)/*dt* = 0 *for t* ∈ [0, *T*] *and some i*, 1 ≤ *i* ≤ *G*, *then*
  $$\begin{array}{c} \frac{W_{i}\left(t\right)}{W_{i,0}}=(\frac{P(t)}{P_{0}}{)}^{k_{i}},\;\forall t\in \left[0,T\right] \end{array}$$
  *In particular*, *if P*(*T*) = *P*_0_ *then W*_*i*_(*T*) = *W*_*i*,0_.
4. *If there are constants α*_1_, *α*_2_, …, *α*_*G*_, *β such that* $\sum_{j=1}^{G}\alpha_{j}k_{j}\left(t\right)=\beta$ *for t* ∈ [0, *T*], *then*
  $$\begin{array}{c} (\frac{W_{1}\left(t\right)}{W_{1,0}}{)}^{\alpha_{1}}(\frac{W_{2}\left(t\right)}{W_{2,0}}{)}^{\alpha_{2}}\cdots (\frac{W_{G}\left(t\right)}{W_{G,0}}{)}^{\alpha_{G}}=(\frac{P(t)}{P_{0}}{)}^{\beta },\;\forall t\in \left[0,T\right] \end{array}$$
5. *If there are nonzero constants η*_1_, *η*_2_, …, *η*_*G*_, *and a function f*(*t*) *such that k*_*i*_(*t*) = *k*_*i*,0_ + *η*_*i*_ *f*(*t*) *for t* ∈ [0, *T*], *then for any i*, 1 ≤ *i* ≤ *G*,
  $$\begin{array}{c} (\frac{W_{i}\left(t\right)}{W_{i,0}}{)}^{\frac{1}{\eta_{i}}}(\frac{P(t)}{P_{0}}{)}^{-\frac{k_{i,0}}{\eta_{i}}}=g\left(P\left(t\right)\right) \end{array}$$
  *where g*(*P*) *is defined implicitly as the function of P that satisfies the following relation* (*for all P*):
  $$\begin{array}{c} \sum_{i=1}^{G}W_{i,0}(\frac{P}{P_{0}}{)}^{k_{i,0}}g{\left(P\right)}^{\eta_{i}}=\bar{M}+\bar{N}P. \end{array}$$
6. *For any i*, *j*, 1 ≤ *i*, *j* ≤ *G*, *the ratio W*_*i*_(*t*)/*W*_*j*_(*t*) *instantaneously increases with t if and only if* $\left(k_{i}\left(t\right)-k_{j}\left(t\right)\right)\frac{dP}{dt}>0$.
7. *If* $\left(\tilde{P}\left(t\right),\tilde{W}\left(t\right)\right)$ *is a solution of* (25)-(26) *with initial conditions P*(0) = *βP*_0_, **W**(0) = *α* **W**_0_, *where α*,*β* > 0, *and with trading rates* $\tilde{k}\left(t\right)=k\left(\sigma \left(t\right)\right)$ *where σ*(*t*) *is a monotone increasing differentiable function*, *then*
  $$\begin{array}{c} \left(\tilde{P}\left(t\right),\tilde{W}\left(t\right)\right)=\left(\beta P\left(\sigma \left(t\right)\right),\alpha W\left(\sigma \left(t\right)\right)\right) \end{array}$$

The proof of Lemma 1 is provided in S2 Text.

In the context of the model, the mathematical statements of Lemma 1 can be interpreted as follows:

1. no investor can gain wealth when price falls or lose wealth when price increases, and the price increases if and only if the direction of the change in trading rates is along the direction of the wealth vector,
2. no investor gains or loses wealth as long as the change in trading rates remains orthogonal to the vector of starting wealth,
3. if any investor pursues the CRP strategy, then his wealth is a monotone function of the price. In particular, after any process in which the price returns to its starting value his terminal wealth equals his initial wealth.
4. when the trading strategies of all investors are constrained by a linear relation, then the wealths of all investors and the price are related by a multiplicative constraint.
5. when the trading strategies of all investors form a linear path in the trading rate space then the wealth of any investor can be expressed as a nonlinear function of the price (a generalization of (iii)).
6. one investor gains wealth relative to another investor if and only if the difference in their trading rates corresponds to the direction of change in price,
7. the dynamics of the system (25)-(26) is rate invariant, and independent of the scaling of the price, and the wealth vector.

## Effects of strategies on investor wealth

We shall now analyze several special scenarios in which the majority of investors pursue CRP strategies and study the gain or loss in wealth of the remaining investors. We begin by focusing on the case in which all investors follow the CRP strategy, which leads to an especially simple market behavior. Then we assume that one or two investors depart from the constant trading rate strategy, pit the participants against each other, and determine the winners and losers.

### All CRP strategies

In the scenario where all investors pursue CRP strategies, the behavior of the system is particularly simple. It follows from (25) that during quasi-steady state dynamics the price stays constant (since the system starts already in equilibrium) and so does the wealth of each of the investors.

### One non-CRP strategy

In the scenario where one investor chooses to pursue a non-CRP strategy while all others pursue CRP strategies, the price no longer remains constant. Intuitively, when the investor chooses to increase his holdings of the asset (i.e., the proportion of his wealth in the asset compared to his total wealth), the price will increase, and so will the wealth of all other investors. However, we show that in this scenario, upon return to the original holdings (i.e., after one period of a cyclic trading strategy) the price returns to its original value and so does the wealth of all investors in the market.

Suppose that all investors pursue CRP strategies except for investor *i* = 1. Using Lemma 1(iii) we can rewrite the system (25)-(26) as a two-variable system:

$$\begin{array}{c} \frac{dP}{dt}=\frac{PW_{1}}{\left(1-k_{1}\right)k_{1}W_{1}+\sum_{i=2}^{G}\left(1-k_{i}\right)k_{i}C_{i}P^{k_{i}}}\frac{dk_{1}}{dt} \end{array}$$ (28)

$$\begin{array}{c} \frac{dW_{1}}{dt}=\frac{k_{1}W_{1}}{P}\frac{dP}{dt} \end{array}$$ (29)

where $C_{i}=W_{i,0}/{P_{0}}^{k_{i}}$. Note that both Eqs (28) and (29) are of the type *dX*/*dt* = *A*(*t*)*dk*_1_/*dt* where *A* is positive. It follows that the functions *P*(*t*) and *W*_1_(*t*) are monotone increasing (decreasing) whenever *k*_1_(*t*) is monotone increasing (decreasing).

Consider now the system:

$$\begin{array}{c} \frac{d\tilde{P}}{d\xi }=\frac{\tilde{P}{\tilde{W}}_{1}}{\left(1-\xi \right)\xi {\tilde{W}}_{1}+\sum_{i=2}^{G}\left(1-k_{i}\right)k_{i}C_{i}{\tilde{P}}^{k_{i}}} \end{array}$$ (30)

$$\begin{array}{c} \frac{d{\tilde{W}}_{1}}{d\xi }=\frac{\xi {\tilde{W}}_{1}}{\tilde{P}}\frac{d\tilde{P}}{d\xi } \end{array}$$ (31)

This system has locally Lipschitz r.h.s everywhere in the positive quadrant, and hence the IVP (30)-(31) with initial conditions $0<\tilde{P}\left(\xi_{0}\right)$ and $0<{\tilde{W}}_{1}\left(\xi_{0}\right)$, 0 < *ξ*_0_ < 1, has a unique solution in that domain. Furthermore, this system has two first integrals which are derived from (27) and take the form:

$$\begin{array}{c} \xi {\tilde{W}}_{1}+\sum_{i=2}^{G}k_{i}C_{i}{\tilde{P}}^{k_{i}}=\bar{N}\tilde{P} \end{array}$$ (32)

$$\begin{array}{c} \left(1-\xi \right){\tilde{W}}_{1}+\sum_{i=2}^{G}\left(1-k_{i}\right)C_{i}{\tilde{P}}^{k_{i}}=\bar{M} \end{array}$$ (33)

These integrals form a system of algebraic equations for $\left(\tilde{P},{\tilde{W}}_{1}\right)$, which has a unique solution $\left(\tilde{P}\left(\xi \right),{\tilde{W}}_{1}\left(\xi \right)\right)$ for each *ξ* in the interval [0, 1].

The following observation follows from the above derivations and from Lemma 1(vii): If $\tilde{P}\left(\xi \right)$ and ${\tilde{W}}_{1}\left(\xi \right)$ are solutions of the IVP (30)-(31) with $\tilde{P}\left(k_{1}\left(0\right)\right)=P_{0}$ and ${\tilde{W}}_{1}\left(k_{1}\left(0\right)\right)=W_{1,0}$, then the functions $P\left(t\right)=\tilde{P}\left(k_{1}\left(t\right)\right)$, $W_{1}\left(t\right)={\tilde{W}}_{1}\left(k_{1}\left(0\right)\right)$ solve the IVP (28)-(29) with initial conditions *P*_0_ and *W*_1,0_. This result has important implications for the change in wealth along *cyclic strategies*, i.e., strategies for which *k*_*i*_(*T*) = *k*_*i*_(0) for some *T* > 0 for *i* = 1, 2, …, *G*. A cyclic strategy represents the case in which an investor temporarily increases (or decreases) his relative investment in the asset before returning to the original proportion. This is commonly done for speculative reasons, in order to take advantage of market fluctuations. A particular case is when the investor enters the market, trades for a finite interval of time, and then exits the market (in this case *k*_1_(*T*) = *k*_1_(0) = 0). The result, summarized in the following theorem, implies that there will be no net change in the investor’s wealth after a cyclic strategy is executed in a quasi-steady state process, provided all other investors pursue CRP strategies.

**Theorem 2**. *Let* (*P*(*t*), **W**(*t*)) *be a solution of the system* (25)-(26) *on the interval* [0, *T*] *with trading rates* **k**(*t*) *and initial conditions P*(0) = *P*_0_, **W**(0) = **W**_0_. *If k*_1_(*T*) = *k*_1_(0) *and dk*_*i*_(*t*)/*dt* = 0 *for t* ∈ [0, *T*], 2 ≤ *i* ≤ *G*, *then P*(*T*) = *P*_0_ *and* **W**(*T*) = **W**_0_.

**Fig 1:**
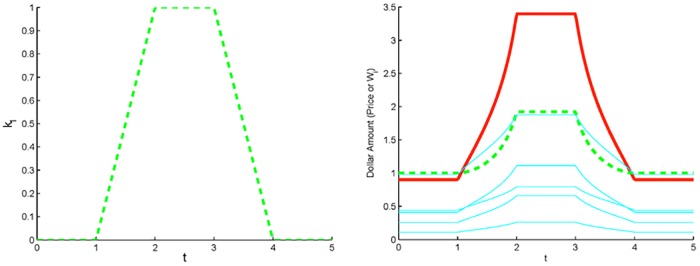
One non-CRP investor. Dynamics of a market with one non-CRP investor who enters and then exits the market in accord with the trading rate *k*_1_(*t*) (panel (a)), and five CRP investors with randomly chosen parameters. In panel (b), the price *P* (solid red), the wealth *W*_1_ (dashed green) of the non-CRP investor, and the wealth of each CRP investor (solid cyan) first increase and then return to their initial values.

In other words, in a quasi-steady process, if one investor executes a cyclic non-CRP strategy while all other investors execute CRP strategies, then the final wealth of every investor equals the starting wealth, and the final price equals the starting price. See Fig 1 for an illustration of this result.

### Two non-CRP strategies

When two investors are executing non-CRP strategies simultaneously, the outcome is different from the previous two cases. As we shall see below, even if the strategies are cyclic, the wealth of both investors can change over one cycle, i.e., one of the investors may gain wealth while the other loses wealth. For example, if one investor becomes aware that another, distressed trader may need to temporarily liquidate his holdings, the first investor can choose to sell before the distressed trader is able to, thereby lowering the price, and subsequently buy back the asset at a lower price. Both traders then execute a cyclic strategy but the first one loses while the second one gains wealth. Another scenario is when information becomes public at time *t*_1_ causing both investors to buy the asset. Subsequently, new information becomes available at time *t*_2_ > *t*_1_ causing both investors to sell. If investor 1 is able to act before investor 2 in both cases, then investor 1 increases his terminal wealth at the expense of investor 2. This type of behavior is called predatory trading [29, 30]. Below we show it is detrimental to the investor’s wealth to be the second-mover or follower of another’s trading strategy (i.e., to not be the first investor to act).

The system with time-dependent trading rates *k*_1_(*t*) and *k*_2_(*t*) while others maintain fixed rates can be analyzed as follows. In view of Lemma 1(iii), (25)-(26) reduce to:

$$\begin{array}{c} \frac{dP}{dt}=\frac{P(W_{1}\frac{dk_{1}}{dt}+W_{2}\frac{dk_{2}}{dt})}{\left(1-k_{1}\right)k_{1}W_{1}+\left(1-k_{2}\right)k_{2}W_{2}+\sum_{i=3}^{G}\left(1-k_{i}\right)k_{i}C_{i}P^{k_{i}}} \end{array}$$ (34)

$$\begin{array}{c} \frac{dW_{1}}{dt}=\frac{k_{1}W_{1}}{P}\frac{dP}{dt} \end{array}$$ (35)

$$\begin{array}{c} \frac{dW_{2}}{dt}=\frac{k_{2}W_{2}}{P}\frac{dP}{dt}. \end{array}$$ (36)

Using the relations (27), we can express *W*_1_ and *W*_2_ in terms of *P* as

$$\begin{array}{c} W_{1}=\frac{P\hat{N}\left(P\right)\left(1-k_{2}\right)-\hat{M}\left(P\right)k_{2}}{k_{1}-k_{2}} \end{array}$$ (37)

$$\begin{array}{c} W_{2}=\frac{\hat{M}\left(P\right)k_{1}-P\hat{N}\left(P\right)\left(1-k_{1}\right)}{k_{1}-k_{2}} \end{array}$$ (38)

with $\hat{N}\left(P\right)=\bar{N}-\sum_{i=3}^{G}k_{i}C_{i}P^{k_{i}-1}$, $\hat{M}\left(P\right)=\bar{M}-\sum_{i=3}^{G}\left(1-k_{i}\right)C_{i}P^{k_{i}}$, and *C*_*i*_ depending on the initial condition. Note that finiteness and positivity of *W*_1_ and *W*_2_ requires that whenever *t* = t* where *k*_1_(t*) = *k*_2_(t*) = k*, then P* = *P*(t*) is determined by k* as the solution of

$$\begin{array}{c} P^{*}\hat{N}\left(P^{*}\right)\left(1-k^{*}\right)=\hat{M}\left(P^{*}\right)k^{*}. \end{array}$$ (39)

Note also that $\partial (P\hat{N}\left(P\right))/\partial P>0$ while $\partial \hat{M}\left(P\right)/\partial P\leq 0$.

Substitution into (34) yields a single equation for *P*:

$$\begin{array}{c} \frac{dP}{dt}=P\frac{P\hat{N}\left(P\right)[\left(1-k_{2}\right)\frac{dk_{1}}{dt}-\left(1-k_{1}\right)\frac{dk_{2}}{dt}]-\hat{M}\left(P\right)(k_{2}\frac{dk_{1}}{dt}-k_{1}\frac{dk_{2}}{dt})}{\left(k_{1}-k_{2}\right)[P\hat{N}\left(P\right)\left(1-k_{1}\right)\left(1-k_{2}\right)+\hat{M}\left(P\right)k_{1}k_{2}+S\left(P\right)]} \end{array}$$ (40)

where $S\left(P\right)=\sum_{i=3}^{G}\left(1-k_{i}\right)k_{i}C_{i}P^{k_{i}}$. Note that the constants *C*_*i*_ in $\hat{N}\left(P\right)$, $\hat{M}\left(P\right)$, and $\hat{S}\left(P\right)$ are determined by the initial conditions (*P*_0_, **W**_0_). Clearly, the Eq (40) is singular whenever *k*_1_(*t*) = *k*_2_(*t*) and hence its solution is not defined at those points. Thankfully, (39) defines the values of *P*(*t*) for all such *t*.

Eq (40) is difficult to solve analytically, but we can obtain information about the behavior of the system by using Lemma 1. First of all, we can deduce from Lemma 1(vii) that the final wealth of each investor after one cycle is independent of how fast the system travels along the cycle, but depends only on the initial conditions (*P*_0_, **W**_0_) of the system and the path *γ* = {**k**(*t*)|*t* ∈ [0, *T*]} of the system in the **k**-space. A cyclic strategy is then represented by an oriented closed path *γ*.

The next theorem relates the changes in *P* and *W*_*i*_ for any cyclic strategy. We have seen in the section above that by maintaining the CRP strategy, the investor minimizes any risk of loss of wealth over a cyclic strategy if one investor in the market does not follow the CRP strategy. Unfortunately, this is no longer true if two or more investors follow time-dependent strategies. In such a situation, at the end of the cycle the price *P* need not return to its original value, and any change in price will result in a change of wealth even for the CRP investors. In addition, between the two investors with non-CRP strategies, one investor’s wealth will increase at the expense of the other.

**Theorem 3**. *Let* (*P*(*t*), **W**(*t*)) *be a solution of the system* (25)-(26) *on the interval* [0, *T*] *with initial conditions P*(0) = *P*_0_, **W**(0) = **W**_0_ *and trading rates* **k**(*t*) *such that dk*_*i*_(*t*)/*dt* = 0 *for t* ∈ [0, *T*], 3 ≤ *i* ≤ *G*, *while k*_1_(*t*) *and* *k*_2_(*t*) *on the interval* [0, *T*] *form a closed curve γ in the* (*k*_1_, *k*_2_) *plane*. *Then for* 3 ≤ *i* ≤ *G*,

$$\begin{array}{ccc} \text{sgn}\left(W_{2}\left(T\right)-W_{2,0}\right) & = & -\text{sgn}\left(W_{1}\left(T\right)-W_{1,0}\right),\\ \text{sgn}\left(W_{i}\left(T\right)-W_{i,0}\right)=\text{sgn}\left(P\left(T\right)-P_{0}\right) & = & \text{sgn}\left(\left(W_{1}\left(T\right)-W_{1,0}\right)\left(k_{1}\left(0\right)-k_{2}\left(0\right)\right)\right) \end{array}$$

The proof of Theorem 3 is provided in S3 Text.

Let us now focus on special paths *γ* for which we can deduce the sign of the change in wealth of investors 1 and 2. Lemma 1(ii) tells us that if a segment of that path is orthogonal to **W** then **W** is constant along that segment. Furthermore, Lemma 1(iv) tells us that along path segments that lie on lines passing through the origin, i.e., for which *α*_1_*k*_1_(*t*) + *α*_2_*k*_2_(*t*) = 0, there is a special relation between *W*_1_(*t*) and *W*_2_(*t*), namely

$$\begin{array}{c} (\frac{W_{1}\left(t\right)}{W_{1,0}}{)}^{\alpha_{1}}=(\frac{W_{2}\left(t\right)}{W_{2,0}}{)}^{-\alpha_{2}} \end{array}$$ (41)

**Fig 2:**
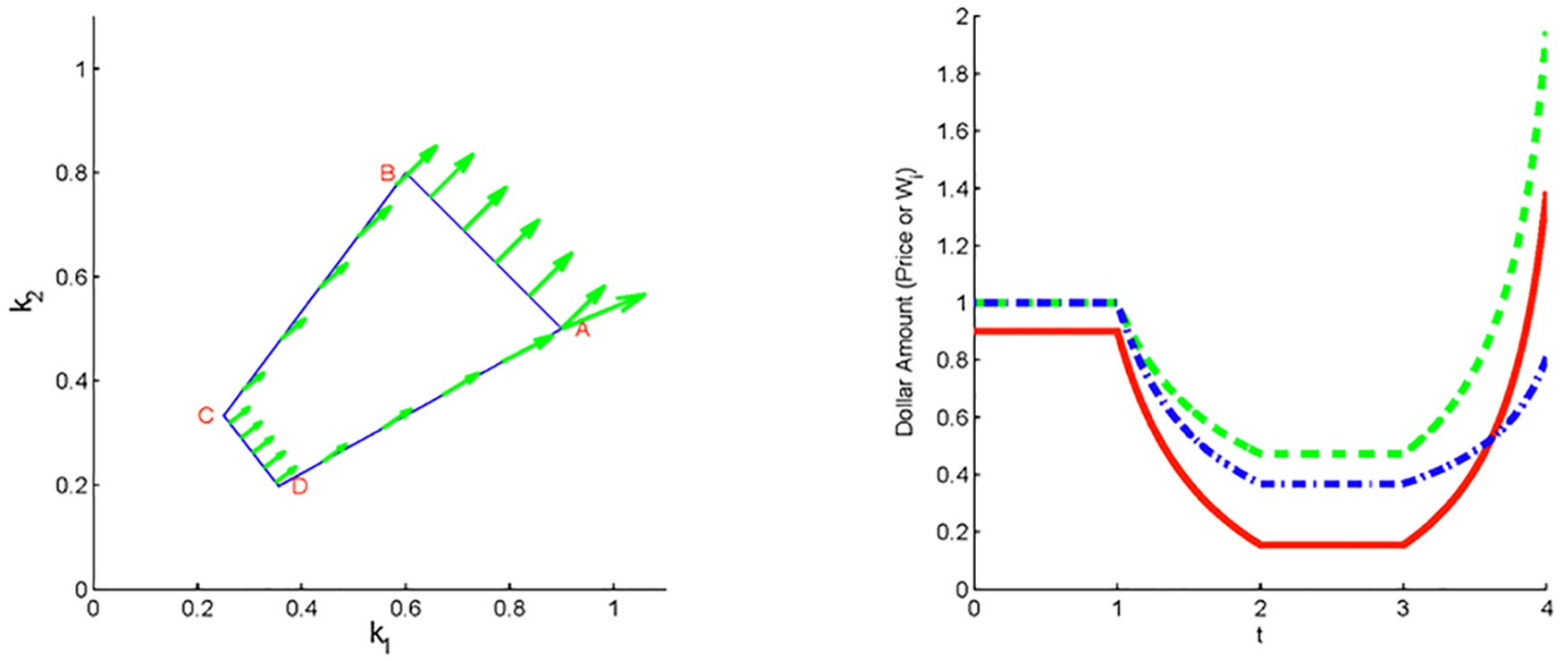
Two non-CRP investors. Dynamics of a market with two non-CRP investors whose strategies follow the closed quadrilateral path in the (*k*_1_, *k*_2_) plane traversed counterclockwise as ABCDA (panel (a)). In panel (b), both the price *P* (solid red) and the wealth *W*_1_ (dashed green) finish above the starting values, while wealth *W*_2_ (dash-dot blue) finishes below. (The green arrows in panel (a) here and in the figures below, depict the vector **W** at selected points along the path.)

Together, we can use these results to construct special strategies corresponding to quadrilateral paths along which *W*_1_ (or *W*_2_) increases (or decreases) during one cycle. Schematic depiction of changes in **W** when investor strategies follow the quadrilateral path ABCD outlined in Lemma 4 are shown in Fig 2.

**Lemma 4**. *Let* (*P*(*t*), **W**(*t*)) *be a solution of the system* (25)-(26) *on the interval* [0, *T*] *with initial conditions P*(0) = *P*_0_, **W**(0) = **W**_0_ *and trading rates* **k**(*t*) *such that dk*_*i*_(*t*)/*dt* = 0 *for t* ∈ [0, *T*], 3 ≤ *i* ≤ *G*, *while k*_1_(*t*) *and k*_2_(*t*) *on the interval* [0, *T*] *form a path in the* **k**-*space that is a counterclockwise labeled quadrilateral ABCD with vertices A* = **k**(0) = **k**(*T*), *B* = **k**(*t*_*B*_), *C* = **k**(*t*_*C*_), *D* = **k**(*t*_*D*_), *where* 0 < *t*_*B*_ < *t*_*C*_ < *t*_*D*_ < *T*, *the side AB is orthogonal to* **W**_0_, *the sides BC and DA lie on distinct lines passing through the origin*, *the side CD is orthogonal to* **W**(*t*_*C*_), *and A is the vertex with the largest k*_1_. *Then W*_1_(*T*) > *W*_1,0_ *and* *W*_2_(*T*) < *W*_2,0_.

The proof of Lemma 4 is provided in S4 Text.

A generalization of Lemma 4 can be obtained for a system that consists of CRP investors and two non-CRP investors who follow an arbitrary piecewise smooth Jordan path:

**Theorem 5**. *Let* (*P*(*t*), **W**(*t*)) *be a solution of the system* (25)-(26) *on the interval* [0, *T*] *with initial conditions P*(0) = *P*_0_, **W**(0) = **W**_0_ *and trading rates* **k**(*t*) *such that dk*_*i*_(*t*)/*dt* = 0 *for t* ∈ [0, *T*], 3 ≤ *i* ≤ *G*, *while k*_1_(*t*) *and k*_2_(*t*) *on the interval* [0, *T*] *form a piecewise smooth Jordan curve γ in the* (*k*_1_, *k*_2_) *plane*. *Then W*_1_(*T*) − *W*_1,0_ > 0 (*and consequently W*_2_(*T*) − *W*_2,0_ < 0) *if and only if γ is traveled counterclockwise*.

The proof of Theorem 5 is provided in S5 Text.

In the context of the model, Lemma 4 and Theorem 5 state that if two investors change their preferences along a non-intersecting curve in the (*k*_1_, *k*_2_) plane then investor 1 gains wealth if the curve is traveled counterclockwise and loses wealth if the curve is traveled clockwise. The result can also be interpreted in the following way: in order to gain wealth, investor 1 must *anticipate* the changes in the strategy of investor 2 so that the peak in *k*_1_(*t*) precedes the peak in *k*_2_(*t*) and the trough of *k*_1_(*t*) precedes the trough in *k*_2_(*t*). In that case, investor 2, whose wealth decreases throughout the process, can also be observed as the follower of the changes in the trading rate of investor 1. This is similar to the predatory trading scenario described in [29] and [30] where investor 1 would be considered the predator and investor 2 the distressed trader.

Theorem 5 has an important consequence that exemplifies the importance of CRP strategies for minimization of investment risks. In an environment consisting of CRP investors, once one investor (say investor 2) departs from the CRP investment strategy, there is a risk that another investor (say 1) may anticipate the strategy of investor 2 and adjust his strategy so as to gain wealth at the expense of investor 2. This can be formalized as:

**Theorem 6**. *Let* (*P*(*t*), **W**(*t*)) *be a solution of the system* (25)-(26) *on the interval* [0, *T*] *with initial conditions P*(0) = *P*_0_, **W**(0) = **W**_0_ *and trading rates* **k**(*t*) *such that dk*_*i*_(*t*)/*dt* = 0 *for t* ∈ [0, *T*], 3 ≤ *i* ≤ *G*. *For any piecewise smooth non-constant cyclic strategy k*_2_(*t*), *t* ∈ [0, *T*], *and any K there exists a cyclic strategy k*_1_(*t*) *with k*_1_(0) = *k*_1_(*T*) = *K such that W*_1_(*T*) − *W*_1,0_ > 0 *and W*_2_(*T*) − *W*_2,0_ < 0.

The proof of Theorem 6 is provided in S6 Text.

The last theorem shows that if a trader deviates from a CRP strategy, then this trader is susceptible to a decrease in wealth should another trader decide to also deviate. Thus, the CRP strategy is rational in that any trader who departs from the CRP strategy can potentially do worse with respect to his change in wealth. Indeed, the CRP strategy is first about minimizing risks and then about maximizing profits.

Before proceeding to the numeral analyses of the model, we summarize the theoretical results of this section. Theorem 2 implies that if all but one investor follow a CRP strategy, then after any cyclic change in the trading rate of the non-CRP investor (and potential temporary increase or decrease of price) both the price and the wealth of all investors return to their initial values. (This describes, for example, the scenario in which a non-CRP investor enters the market, trades the asset for a while, and then sells the asset and exits the market.) Theorems 3, 5, and 6 state that if two investors follow cyclic non-CRP strategies, then (i) wealth will be transfered from one investor to the other, (ii) the direction of the wealth transfer benefits the investor who pre-empts the other’s strategy, and (iii) every non-CRP strategy can lead to a loss of wealth by the choice of an appropriate competing non-CRP strategy (i.e., no non-CRP strategy is safe). If the price changes during this process, then the terminal wealths of the CRP traders will also change. However, as our numerical studies shown below indicate, the changes in wealth of the CRP investors are order of magnitude smaller than those of the non-CRP investors. From a game-theoretic perspective, Theorem 6 suggests that the CRP strategy minimizes risk among all potential investor strategies, and hence is a pure strategy Nash equilibrium of the system when considered as a differential game with wealth gain representing the payoff.

## Numerical results

Here we present numerical studies of various trading scenarios illustrating the results in the previous sections and a few additional observations. Our primary objective is the analysis of the dynamics of traders’ wealth using the scenario in which all traders adhere to CRP strategies as the baseline. We then consider the impact to investors’ wealth positions as certain investors begin to follow non-CRP strategies. The initial wealth of each non-CRP investor has been scaled to one to facilitate interpretation of the economic meaning of the graphical/numerical results presented in this section. Moreover, the percentage change in wealth (as well as price) is included in Table 3.

As our first example, let us consider a scenario in which a non-CRP investor joins a market consisting of five CRP investors, trades for a set period of time, and then exits the market. (Note that the trading rate functions, *k*_*i*_(*t*), are assumed to be continuous. Discontinuous trading rates will be discussed in a future study.) This represents a cyclic strategy for the single non-CRP investor as the initial trading rate, *k*(0) = 0, equals the ending trading rate, *k*(*T*) = 0, where *T* is the period of one cycle (see Fig 1(a)). Thus, Theorem 2 applies and the terminal wealth should equal the initial wealth for each investor in the system. Indeed, consider Fig 1(b). The wealth curves for the CRP investors (thin lines) follow the relation, $W_{i}\left(t\right)=C_{i}P{\left(t\right)}^{k_{i}}$, given in Lemma 1(iii). Note that *C*_*i*_, *i* = 2, …5, was randomly chosen in the simulations. The ending price *P*(*T*) is equal to *P*_0_, and *W*_*i*_(*T*) are equal to *W*_*i*,0_, to within the accuracy of numerical simulations. Not surprisingly, the main result of the addition of a single non-CRP investor to a market of CRP investors is an increase in demand for the asset which leads to an increase in price by a factor of 3.5, amounting to a price “bubble” as the non-CRP investor increases his holdings in the asset. The price maintains its high level until the non-CRP investor begins his exit from the market. [12] conjecture bubbles may result from the actions of momentum traders with bubbles cresting when these traders run out of cash. Here we see the occurrence of a bubble when a single investor enters the market. In both scenarios there is an influx of cash driving the bubble—consistent with theoretical [9], experimental [15, 32], and empirical [33] studies.

As our second example, let us consider a scenario with two investors who follow trading preferences along the polygonal curve depicted in Fig 2(a), which satisfies the assumptions of Theorem 4. Indeed, the curve is traveled counterclockwise starting at A. Sides BC and DA lie on distinct lines through the origin, while side AB is perpendicular to **W**(*t*_0_) = (*W*_1,0_, *W*_2,0_) = (1, 1), and side CD is perpendicular to *W*(*t*_*C*_). The green arrows represent the vector (*W*_1_, *W*_2_) at selected points along the strategy path. Note that in accord with Lemma 1(ii), **W** is constant along AB and along CD, while along BC and along DA, **W** rotates clockwise in accord with Lemma 1(iv). As a result, there is a net change in *W*_1_ and *W*_2_ along the cycle, i.e., *W*_1_(*T*) > *W*_1,0_ and *W*_2_(*T*) < *W*_2,0_, as indicated by two arrows at the starting point A corresponding to **W**(0) and **W**(*T*). Fig 2(b) displays plots of the price and the wealths of both investors versus time.

**Fig 3:**
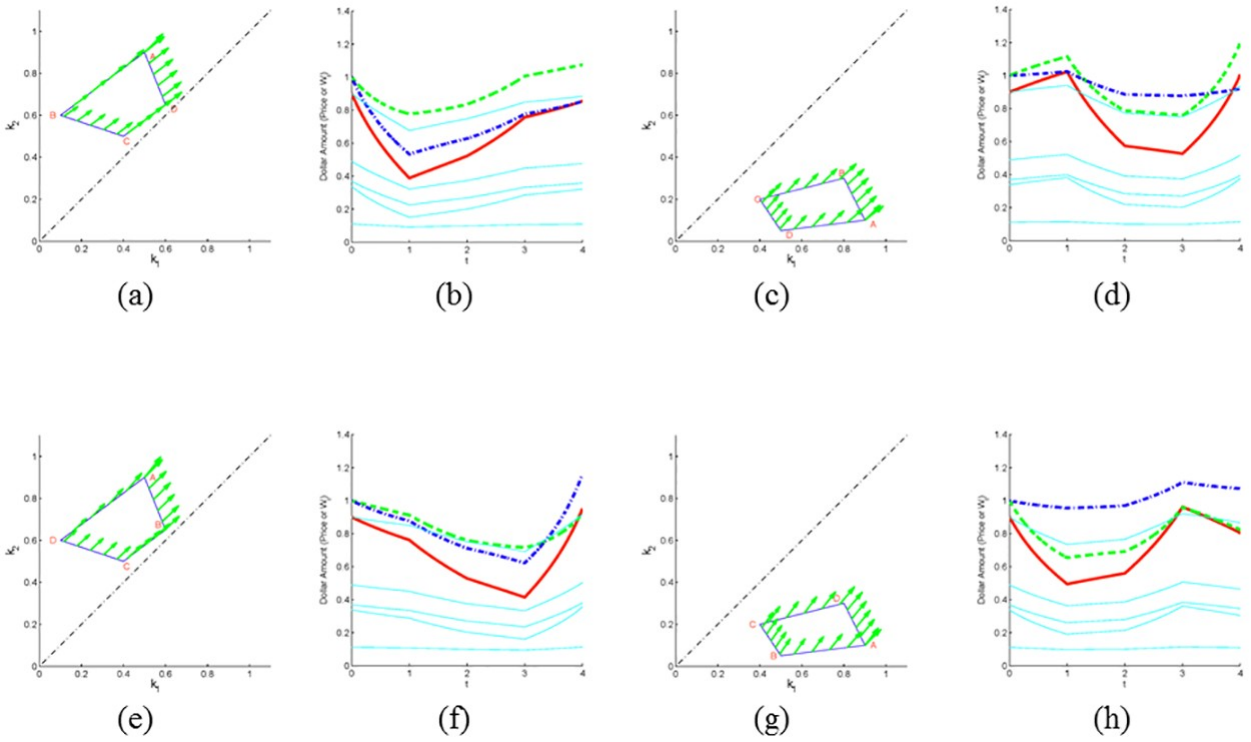
Two non-CRP investors. Dynamics of a market with two non-CRP and five CRP investors (with randomly chosen parameters). The non-CRP investors 1 and 2 follow strategies corresponding to the closed paths (traversed as ABCDA) in panels (a), (c), (e), and (g), and give rise to dynamics in panels (b), (d), (f), and (h), respectively. In (b) and (d), the wealth *W*_1_ (dashed green) finishes above its starting value and *W*_2_ (dash-dot blue) finishes below (since the path is traveled counterclockwise), while in (f) and (h) the situation is reversed. The wealth of the CRP investors (solid cyan) is essentially unchanged. The price (solid red) finishes above its starting value in (d) and (f) and below in (b) and (h).

In the next set of examples, we consider the scenarios corresponding to a market with seven traders of which two follow cyclic trading strategies, while the rest follow CRP strategies. The closed strategy paths in Fig 3(a) and 3(c) are traversed counterclockwise (ABCDA) with *k*_1_(*t*) < *k*_2_(*t*) in Fig 3(a) and *k*_1_(*t*) > *k*_2_(*t*) in Fig 3(c). The curves in Fig 3(e) and 3(g) are the same as in 3(a) and 3(c), but traversed clockwise (ABCDA) with *k*_1_(*t*) < *k*_2_(*t*) in Fig 3(e) and *k*_1_(*t*) > *k*_2_(*t*) in Fig 3(g). Fig 3(b), 3(d), 3(f) and 3(h) display the corresponding evolution of the price and wealths of investors one through seven. Investors 3 through 7 adhere to CRP strategies with parameters that were chosen randomly and kept identical for all path choices in Fig 3. The results shown in these figures are consistent with the results of Theorem 3, in that the price (and consequently the wealth of all CRP investors) decreases along the cycle if the path is traveled counterclockwise and *k*_1_(*t*) < *k*_2_(*t*), or if the path is traveled clockwise and *k*_1_(*t*) > *k*_2_(*t*). Incidentally, in this example all curves are also Jordan curves hence the outcome is consistent with the results of Theorem 5, in that *W*_1_(*T*) > *W*_1,0_ for curves traveled counterclockwise and *W*_1_(*T*) < *W*_1,0_ for those traveled clockwise.

**Fig 4:**
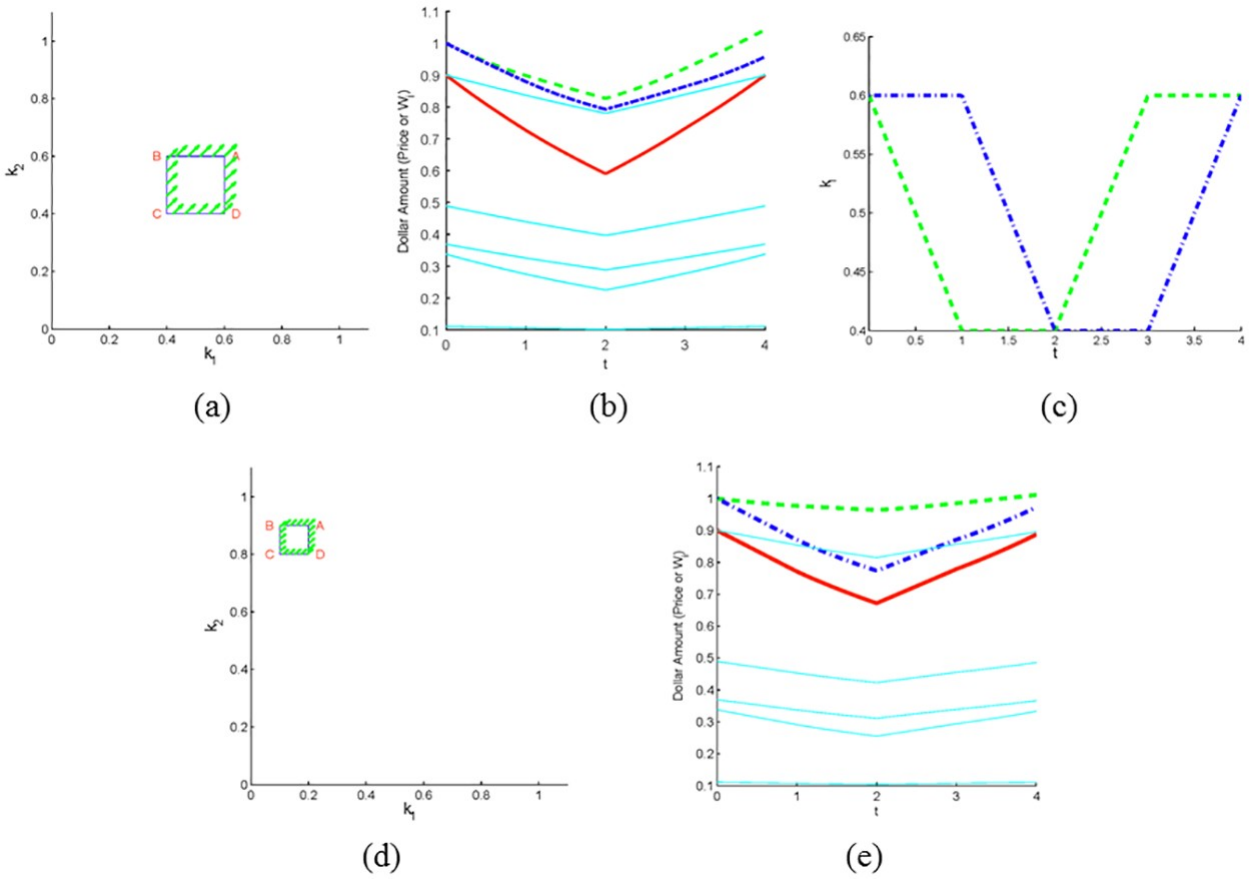
One non-CRP investor gains at the expense of a second non-CRP investor. Dynamics of a market with two non-CRP investors that follow strategies in panels (a) and (d) (traversed counterclockwise as ABCDA). In panels (b) and (e), the wealth *W*_1_ (dashed green) finishes above its starting value while the wealth *W*_2_ (dash-dot blue) finishes below. The price (solid red) and the wealth of CRP investors (solid cyan) are essentially unchanged. Panel (c) shows how in panel (a) the trading rate *k*_2_ of investor 2 (dash-dot blue), follows with a delay that of investor 1, *k*_1_ (dashed green).

In the next set of figures, let us look more closely at scenarios in which investor 1 anticipates the actions of investor 2. This scenario is an appropriate idealization of cases in which a news announcement is made at time *t* = 0 causing both investors to sell the asset, but investor 1 is able to act more quickly than investor 2. Thus, investor 1 reduces his position in the asset (from 60% of his wealth to 40%) from time *t* = 0 to *t* = 1, i.e. along the line segment AB in Fig 4(a). At this point investor 2 begins to sell, while investor 1 maintains his position. Next, suppose another piece of information becomes available at time *t* = 2, point C, causing the investors to buy. Again, investor 1 is able to react more quickly (along line segment CD), while investor 2 does not begin to buy until time *t* = 4, point D. While the investors begin and end with the same strategy in the (*k*_1_, *k*_2_) plane, point A, and have the same initial wealth, *W*_*i*_ = 1, the ending wealth of investor 1 has increased, while that of investor 2 has decreased (see Fig 4(b)). By pre-empting the moves of his fellow investor, investor 1 gains wealth (i.e., *W*_1_(*T*) > *W*_1,0_) at the expense of investor 2. As noted in the prior section, the peak (trough) in *k*_1_(*t*) precedes the peak (trough) in *k*_2_(*t*). This is depicted graphically in Fig 4(c). The trough for investor 2 occurs during 2 ≤ *t* ≤ 3, while the trough for investor 1 occurs for 1 ≤ *t* ≤ 2. Similarly, the peak for investor 2 begins at *t* = 4, while the peak for investor 1 occurs from *t* = 3 to *t* = 4. Note that the scenarios depicted in Fig 4 are similar to the predatory trading scenario described in [29] and [30]. In particular, Fig 4 is analogous to Figs 1 and 2 in [29].

As follows from Theorems 3 and 5 and is depicted in Fig 3, if all but two investors (say investors 1 and 2) utilize CRP strategies, then *W*_*i*_(*T*) < *W*_*i*,0_, *i* ≥ 3, provided *k*_1_(0) < *k*_2_(0) and the curve in (*k*_1_, *k*_2_) plane is traversed counterclockwise. Interestingly, note that investor 1 need not hold a large position in the asset in order to negatively impact the price and subsequently the wealth of all other investors. Indeed, consider Fig 4(d) and 4(e) in which investor 1 begins with 20% of his wealth in the asset, reduces to 10%, and ends again with 20%. In this scenario the wealths of investors 2 through 7 decrease by an average of 2%, while the price drops 3.2%.

It is interesting to note that the inclusion of additional CRP traders acts as a dampener on the system. That is, as the number of CRP investors increases, the wealth changes for all investors (including the non-CRP investors) decrease. Using the scenario of Fig 4(b) as an example, if we fix the strategies of non-CRP investors 1 and 2, and change the number of CRP investors to 0, 1, 2, 5, 10, 20, or 50, then the change in wealth of investor 1 is 8.12%, 6.49%, 6.37%, 4.22%, 3.00%, 1.75%, 0.87%, respectively. This follows from equation (34) that models the change in price. The numerator is a sum over the two non-CRP investors, while the denominator is a sum over all investors. Thus, as the number of CRP investors grows, the instantaneous change in price decreases. And, equations (35) and (36) imply that instantaneous changes in the wealth of the non-CRP investors is proportional to the change in price. From an economics perspective, CRP investors will sell (buy) to rebalance their portfolio in response to an increase (decrease) in price. This, of course, exerts a downward (upward) pressure on the price thereby mitigating the price change. As the number of CRP investors in the market increases, this effect will be magnified resulting in smaller and smaller price changes. Recall that non-CRP investor 1 increases his wealth by preempting the trading strategy of non-CRP investor 2. Larger price changes correspond to larger gains (losses) for investor 1 (2). Thus, the inclusion of additional CRP investors decreases the potential for gains by investor 1.

**Fig 5:**
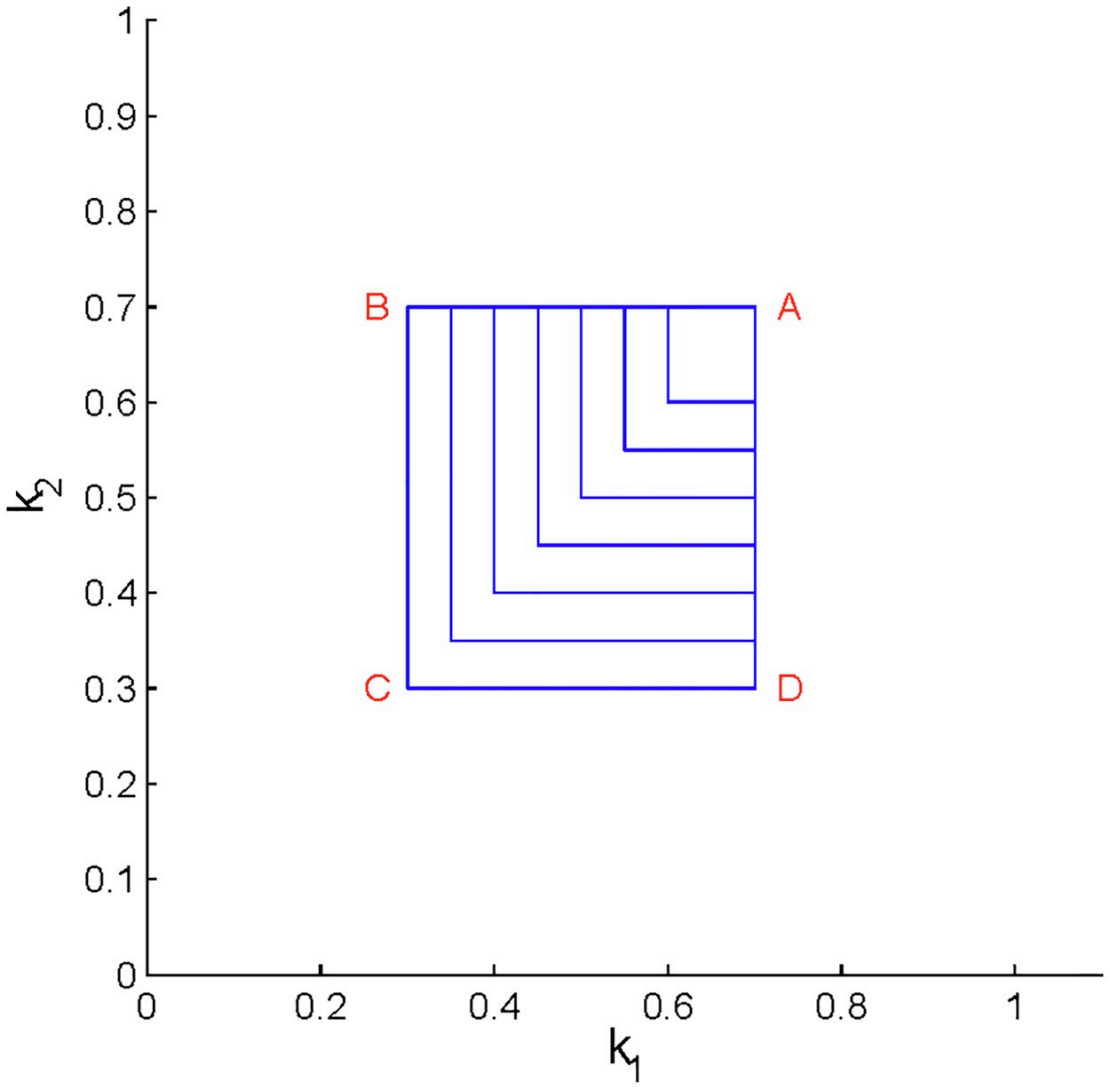
Two non-CRP investors’ wealth along inscribed squares. Seven square paths in the (*k*_1_, *k*_2_) plane. The largest square, *S*_1_, is labeled ABCD.

In the next example we explore the effect of the area enclosed by the path in (*k*_1_, *k*_2_) plane on the magnitude of wealth increase/decrease along the path. We performed seven simulations of a market with seven investors consisting of two non-CRP investors (1 and 2) and five CRP investors. In each simulation the strategies of investors 1 and 2 vary along a counterclockwise square path labeled *S*_*i*_ (here *S*_1_ denotes the square with the largest area and *S*_7_ the square with the smallest area) with starting vertex A = (0.7, 0.7) (see Fig 5). Investors 1 and 2 each begin with an initial wealth of 1, and the starting price is 0.9. The initial wealths and strategies for investors 3-7 were randomly chosen and then used for each of the seven simulations. Consistent with Theorem 5, the relative change in wealth is positive for investor 1 and negative for investor 2. Moreover, note that the magnitude of relative change in wealth decreases for both investors as the area of the square traversed decreases (see Table 1). Since *k*_1_(0) = *k*_2_(0) in this scenario, in accord with Theorem 3, investor 1’s increase in wealth comes solely at the expense of investor 2, and there is no difference between the beginning and ending price.

**Table 1:** Relative change in wealth of non-CRP investors 1 and 2 along each square path in Fig 5.

Investor	*S*_1_	*S*_2_	*S*_3_	*S*_4_	*S*_5_	*S*_6_	*S*_7_
*One*	0.1668	0.1269	0.0929	0.0645	0.0414	0.0235	0.0106
*Two*	-0.1.668	-0.1269	-0.0929	-0.0645	-0.0414	-0.0235	-0.0106

The area of the square decreases from *S*_1_ to *S*_7_.

All preceding examples describe scenarios in which the strategies of two investors form non-intersecting paths in the (*k*_1_, *k*_2_) plane, with the possible addition of several CRP investors with constant *k*_*i*_. In the next set of examples we explore the dynamics of a market with a total of seven investors, in which a cyclic strategy path of investors 1 and 2 intersects itself once forming two closed “loops.” Again, the parameters for CRP investors 3 through 7 were randomly chosen and then the same values were utilized in each simulation.

**Fig 6:**
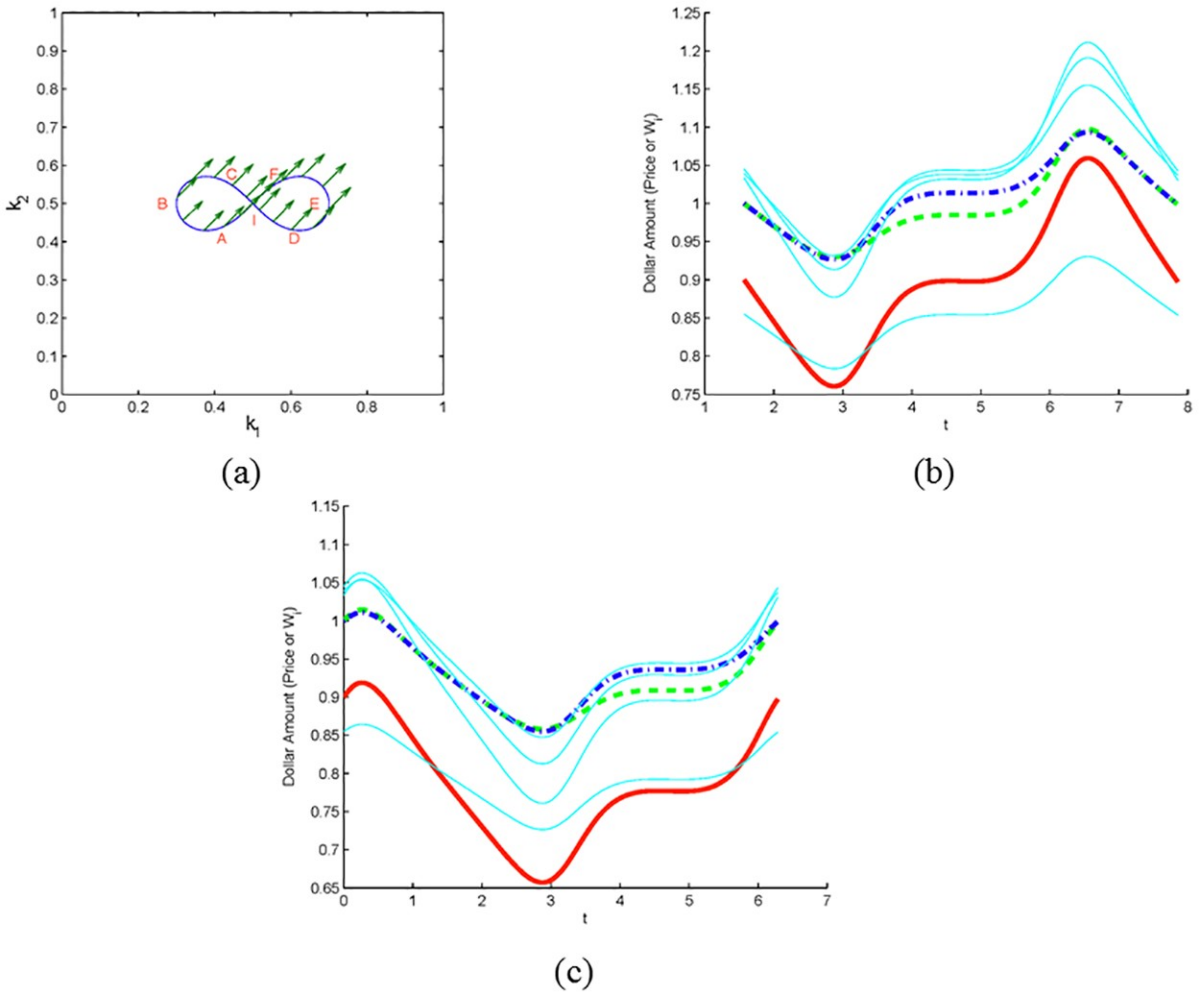
Two non-CRP investors’ strategies corresponding to a lemniscate. Dynamics of a market with two non-CRP investors and five CRP investors with randomly chosen parameters. Investors 1 and 2 follow strategies corresponding to the lemniscate path in panel (a). For strategies corresponding to the path traveled as IABCIDEFI (b) or as EFIABCIDE (c), the wealth *W*_1_ (dashed green), the wealth *W*_2_ (dash-dot blue), the price (solid red), and the wealth of CRP investors (solid cyan) return essentially to their starting values.

First we consider a symmetric lemniscate path in which the two loops (IABCI and IDEFI) enclose equal areas, shown in Fig 6(a). The remaining panels of this figure show that the evolution of price and wealths of all investors is strongly dependent upon the starting point on the path. The dynamics shown in Fig 6(b) pertains to the case in which the lemniscate path starts and ends at the intersection point I, traces the “left-hand” loop in a clockwise direction (IABCI), and then follows the “right-hand” loop in a counterclockwise direction (IDEFI). As Table 2 shows, after completing the first loop, the wealth of investor 2 has increased at the expense of investor 1, and there is no change in price, consistent with Theorems 3 and 5. As the second (“right-hand”) loop is traversed in a counterclockwise manner, the wealth of investor 1 increases while the wealth of investor 2 decreases, and there is no change in price (again consistent with Theorems 3 and 5 applied to the path IDEFI). The increase in *W*_1_ in the second loop is not large enough to make up for the decrease in *W*_1_ in the first loop, and hence the overall result is a decrease in wealth of investor 1 when the complete lemniscate (IABCIDEFI) is traveled. The dynamics shown in Fig 6(c) are produced by following the lemniscate in the same direction, but starting and ending at the apex E of the right loop. (Note that the same initial wealths, price, and *k*_*i*_ values were utilized to produce the figures in panels (b) and (c).) Although the ending wealths and price are similar as in the previous situation (see the second and third panels in Table 2), they are not identical, since the starting point E is such that *k*_1_(0)>*k*_2_(0) and, in accord with Theorem 3, price decreases during the process. Therefore, the choice of the starting point for the excursion around the strategy curve has significant effect on the dynamics of the system.

**Table 2:** Changes in price and investor wealth.

	*P*	*W*_1_	*W*_2_	*W*_3_	*W*_4_	*W*_5_	*W*_6_	*W*_7_
Initial	0.9000	1.0000	1.0000	0.8551	1.0334	1.0391	1.0452	0.9870
**Left-hand loop IABCI**								
Δ	0	-0.0147	0.0147	0	0	0	0	0
**Lemniscate path IABCIDEFI**								
Δ(×10^3^)	0	-0.156	0.156	0	0	0	0	0
**Lemniscate path EFIABCIDE**								
Δ(×10^3^)	-0.020	-0.168	0.141	-0.010	-0.023	-0.015	-0.019	-0.010
**Self-intersecting curve ABICDEIFA**								
Δ	-0.0013	-0.0199	0.0183	-0.0007	-0.0015	-0.0010	-0.0012	-0.0007

**Fig 7:**
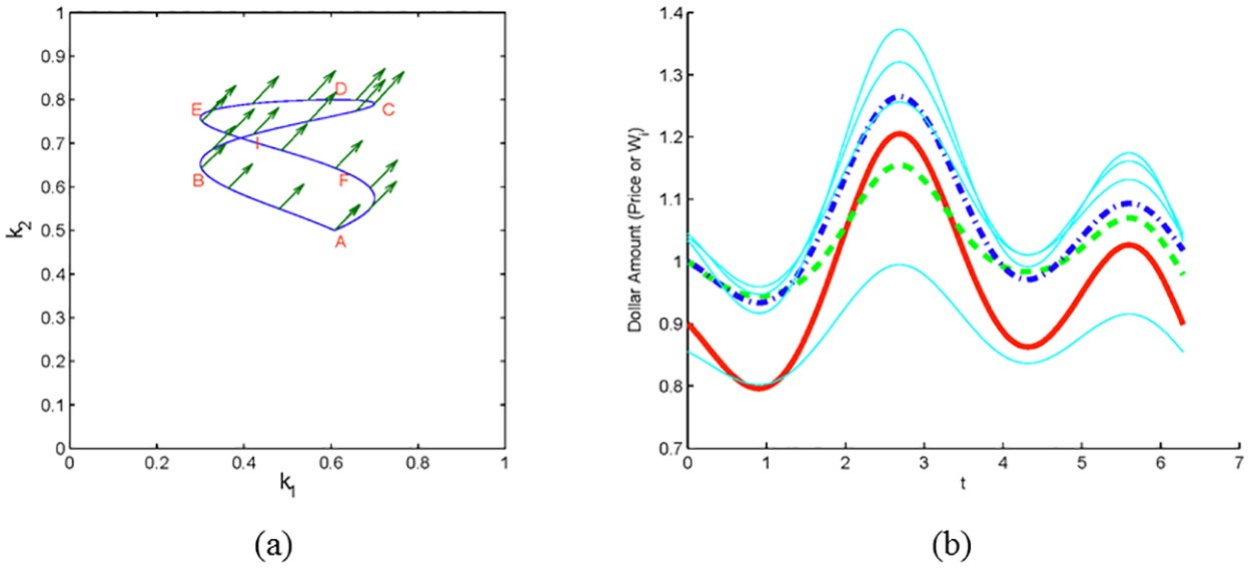
Two non-CRP investors’ strategies corresponding to a non-symmetric lemniscate. Dynamics of a market with two non-CRP investors and five CRP investors with randomly chosen parameters. The non-CRP investors 1 and 2 follow strategies corresponding to the path in panel (a) traversed as ABICDEIFA. In panel (b) the price (solid red), the wealth *W*_1_ (dashed green), and the wealth of CRP investors (solid cyan) finish below their starting values, while the wealth *W*_2_ (dash-dot blue) finishes above.

Changes in price and the wealth of the non-CRP investors (1 and 2) and the CRP investors (3 through 7) corresponding to one excursion around the curves defined in Figs 6 and 7. The initial price and wealth are chosen to be the same for each scenario.

In the next example we consider a self-intersecting path that is not symmetric and which has two loops (IFAB and ICDE) that enclose unequal areas (see Fig 7(a)). We start at the point A and move to the left passing through the points BICDEIF and returning to A. Note the oscillatory behavior of the price and wealth curves in panel (b). Although the ending wealths are similar to the initial wealths (see the bottom panel of Table 2), investor 1 loses while investor 2 gains wealth, which is consistent with the fact that the loop that is traveled clockwise (IFABI) is larger in area than that traveled counterclockwise (ICDEI). Note that the wealth of each investor (whether a CRP or a non-CRP investor) may vary dramatically throughout the timeframe.

**Fig 8:**
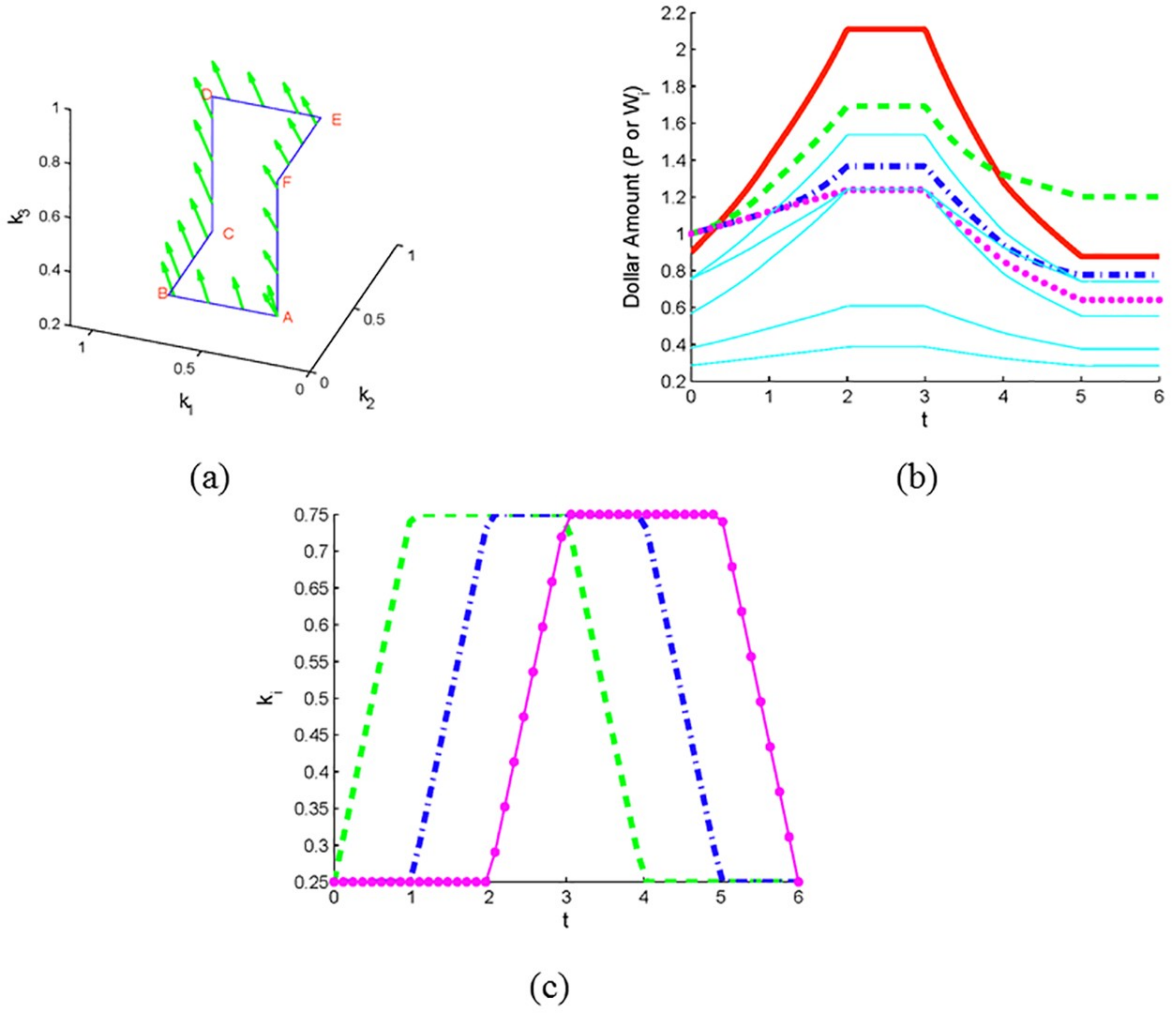
Three non-CRP investors. Dynamics of a market with three non-CRP investors and five CRP investors with randomly chosen parameters. The non-CRP investors 1, 2, and 3 follow strategies corresponding to the polygonal path in panel (a), which is traversed as ABCDEFA. Panel (b) shows the price (solid red curve), *W*_1_ (dashed green), *W*_2_ (dash-dot blue), *W*_3_ (magenta circles), and the wealth of CRP investors (thin solid cyan). Panel (c) shows investors’ trading rates versus time. Note that investor 2’s actions follow those of investor 1 and investor 3’s actions follow those of investor 2.

The last example describes a market with three non-CRP investors following cyclic trading strategies where all three investors react to information in the same manner (i.e., they temporarily alter their trading preferences *k*_*i*_), but investor 1 acts first followed by investor 2 and then by investor 3 (see Fig 8(a) which is traversed as ABCDEFA). In Fig 8(b), similar to Fig 4, one can also observe a transfer of wealth: after one complete cycle, only investor 1 experiences an increase in wealth, while investors 2 and 3 both have a decrease in wealth. Moreover, the wealth of investor 3 (the last investor to act) declines more than that of investor 2. The curves in panel (c) of Fig 8 also demonstrate that investor 1 is the first to act, while investor 3 is the last to act.

To facilitate interpretation of the above results as well as clearly identify their economic significance, we present the percentage change in price and wealth of each investor in Table 3.

**Table 3:** Percentage change in price and wealth of each investor for Figs 1, 2, 3, 4, 6, 7, 8.

		non-CRP			CRP				
Fig	*P*	*W*_1_	*W*_2_	*W*_3_	*W*_4_	*W*_5_	*W*_6_	*W*_7_	*W*_8_
1	0.000%	0.000%			0.000%	0.000%	0.000%	0.000%	0.000%
2	54.40%	95.20%	-19.04%						
3*b*	-5.100%	7.550%	-14.630%		-2.580%	-4.900%	-1.770%	-3.020%	-1.170%
3*d*	11.68%	19.78%	-7.820%		5.660%	11.19%	3.830%	6.680%	2.500%
3*f*	5.560%	-8.080%	15.94%		2.740%	5.330%	1.860%	3.220%	1.220%
3*h*	-10.54%	-17.63%	7.350%		-5.400%	-10.14%	-3.720%	-6.310%	-2.460%
4*b*	0.000%	4.220%	-4.220%		0.000%	0.000%	0.000%	0.000%	0.000%
4*e*	-1.420%	1.130%	-2.690%		-0.710%	-1.360%	-0.490%	-0.830%	-0.320%
6*b*	0.000%	-0.016%	0.016%		0.000%	0.000%	0.000%	0.000%	0.000%
6*c*	-0.002%	-0.017%	0.014%		-0.001%	-0.002%	-0.001%	-0.002%	-0.001%
7	-0.150%	-1.990%	1.830%		-0.080%	-0.150%	-0.100%	-0.120%	-0.070%
8	-2.600%	20.11%	-22.23%	-35.97%	-0.920%	-2.160%	-1.530%	-1.440%	-2.380%

Percentage changes in price and the wealth of investors following either non-CRP or CRP strategies.

## Conclusion

We have utilized the multi-group asset flow model of Caginalp and collaborators to examine the performance of CRP strategies and to better understand the wealth dynamics of both CRP and non-CRP investors. Similar to the Merton (1971) framework, the asset flow equations we utilize here form a continuous time model with no transaction costs. We find that if all investors adhere to CRP strategies, then the wealth of each investor along with the price is constant. If, however, one investor follows a non-CRP strategy, while all other investors utilize CRP strategies, then the wealth of each investor as well as the price is no longer constant. Although the ending wealth (i.e., wealth at the end of each period of the non-CRP investor’s strategy) of all investors equals the initial wealth, provided that the non-CRP investor follows a cyclic strategy, the wealth trajectories of all investors are not constant. Indeed, the strategy of the non-CRP investor affects the demand for the asset and may result in a price bubble or a drop in the price and consequently a rise or a drop in the wealth of each investor.

As noted in the Introduction, several studies have considered the impact of heterogeneous investor beliefs on price and wealth dynamics via deterministic pricing models. Here we abstract away from the specific motivation (classical or behavioral) to focus on the impact of time-dependent trading strategies. In the baseline case, constant trading strategies correspond to CRPs. Then, by allowing the trading strategy to vary over time, we are able to consider the wealth dynamics of various scenarios. For example, can a trader with a time dependent strategy increase his/her wealth when all other traders adhere to CRP strategies? Alternately, suppose an investor plans to enter the market for a period of time and then exit the market. Within the realm of CRP strategies, are there situations in which this investor can be assured of not losing wealth? The value of our results is two-fold. First, we provide rigorous mathematical support and explanation for some commonly used strategies and intuitive investment practices, and second, we provide a framework in which more complicated relations of many investment strategies can be evaluated and their outcomes described in a quantitative fashion.

Our results compare to (and are consistent with) those of previous studies. Indeed, [28] provides a robust summary of the literature regarding the 1/*n* strategy and points out that: (i) the 1/*n* strategy is difficult to outperform in a volatile market and (ii) behavioral studies note the prevalent intuitive use of 1/*n* strategies in a variety of settings. With respect to the former point, the authors also show that the 1/*n* strategy is a rational choice when the investor is faced with a high degree of model uncertainty. In [34], the authors perform an empirical study using monthly data for the 30 Fama-French industry portfolios. Employing an out-of-sample analysis to compare the performance of the 1/*n* strategy to the Mean-Variance efficient portfolio with the same level of risk, they find that the 1/*n* strategy outperforms the Mean-Variance portfolio for smaller portfolios (*n* < 30), while the Mean-Variance portfolio outperforms for larger (*n* >= 30) portfolios. In [35] the 1/*n* rule was used as a benchmark against which 14 other trading strategies were tested. The authors found that none of these more “sophisticated” strategies, including a “minimum variance” portfolio strategy in which the only objective was to minimize the variance of returns (i.e., risk), consistently outperforms the 1/n strategy with respect to the Sharpe ratio, certainty-equivalent return, or turnover. These results, which relate a version of the CRP strategy to risk minimization, are similar to the main takeaway of Theorem 6.

We have limited our study to cases in which the strategies of all investors are constant or periodic with commensurate period. This was done because we are interested in the differences in investor wealth arising from the investors’ use of distinct trading strategies and not from an overall increase/decrease in the market price. That is, we focus on the relative transfer of wealth between investors (due to their choice of strategy) and not the wealth trends common to all investors. Our results can be generalized to scenarios in which price, and consequently the wealth of all investors, increases monotonically due to a steady change in demand or supply.

The model can also handle strategies such as the (all-in) “buy and hold” strategy (*k*_*i*_(*t*) = 1) as well as a complete exit from the market (*k*_*i*_(*t*) = 0). It is fairly common for an investor to use a portion of his cash to buy shares of the asset and then simply hold them along with the remainder of his cash. We consider this to be a *partial* buy and hold strategy, which may be treated as a mixed strategy with *k*_*i*_(*t*) = 1 applied to part of the investor’s holdings and *k*_*i*_(*t*) = 0 to the rest. As such, the strategies for both parts of the investor’s holdings fall under the category of CRP strategies and our results apply.

Our main result considers two investors (identified as investors 1 and 2) who follow cyclic non-CRP strategies corresponding to a Jordan curve in the (*k*_1_, *k*_2_) strategy plane. In that case, if the curve is traversed in the counterclockwise direction, the ending wealth of investor 1 increases (primarily) at the expense of investor 2. This suggests that if investor 1 is able to anticipate the action of investor 2, then he may profit at the expense of investor 2. In plain words, this result corresponds to the commonly accepted notion that it is better to be the first mover rather than a follower when reacting to trading news/activities. It is noteworthy that in the case of two non-CRP investors any CRP investors may gain or lose wealth. Numerical simulations, though, suggest these losses (or gains) are much smaller than those incurred by the “trailing” non-CRP investor.

In this study we assume (i) the investors’ trading strategies, *k*_*i*_(*t*), are continuous and (ii) the traders’ actions influence the price. Moving forward, we intend to examine the wealth dynamics when these assumptions are relaxed. Indeed, suppose the trading strategies were piecewise-constant instead of continuous. This might be a truer reflection of actual trading strategies, where investors update their portfolios on a periodic basis. In addition, suppose the traders in the market are “small” in the sense of [36], i.e., the traders’ actions do not impact the price. As [37] find that market depth has increased over the years both at and behind the NBBO (national best bid and offer), it is reasonable to consider scenarios in which traders are price takers. That is, for a majority of small investors trades have minimal (if any) effect on market prices. Consider, for example, traders with small(er) positions trading in highly liquid securities (e.g., Dow Jones stocks).

## Supporting information

S1 Text Derivation of *dP*/*dt*. Here we provide the derivation of the formula (25) for *dP*/*dt*.
(PDF)

S2 Text Proof of Lemma 1. Here we present the statement and proof Lemma 1.
(PDF)

S3 Text Proof of Theorem 3. Here we present the statement and proof of Theorem 3.
(PDF)

S4 Text Proof of Lemma 4. Here we present the statement and proof of Lemma 4.
(PDF)

S5 Text Proof of Theorem 5. Here we present the statement and proof of Theorem 5, and the statement and proof of a lemma utilized in the proof of that theorem.
(PDF)

S6 Text Proof of Theorem 6. Here we present the statement and proof of Theorem 6.
(PDF)

S7 Text Matlab code to create Fig 1. Here we provide a Matlab program to produce Fig 1 as well as generate data for Table 3.
(M) Click here for additional data file.

S8 Text Matlab code to create Figs 2, 3 and 4 and Table 1. Here we provide a Matlab program to produce Figs 2, 3, and 4 as well as generate data for Tables 1 and 3.
(M) Click here for additional data file.

S9 Text Matlab code to create Fig 4. Here we provide a Matlab program to produce Fig 4 with different numbers of CRP investors (e.g., 0, 1, 2, 5, 10, 20, or 50).
(M) Click here for additional data file.

S10 Text Matlab code to create Fig 5. Here we provide a Matlab program to produce Fig 5.
(M) Click here for additional data file.

S11 Text Matlab code to create Fig 6 and Table 2. Here we provide a Matlab program to produce Fig 6 as well as generate data for Tables 2 and 3.
(M) Click here for additional data file.

S12 Text Matlab code to create Fig 7 and Table 2. Here we provide a Matlab program to produce Fig 7 as well as generate data for Tables 2 and 3.
(M) Click here for additional data file.

S13 Text Matlab code to create Fig 8. Here we provide a Matlab program to produce Fig 8 as well as generate data for Table 3.
(M) Click here for additional data file.
