# Supplementary material for: Slow-fast analysis of a multi-group asset flow model with implications for the dynamics of wealth
Source: PLoS One. 2018 Nov 29;13(11):e0207764. doi: 10.1371/journal.pone.0207764 (PMC6264481; doi:10.1371/journal.pone.0207764)
Supplement: S1 Text — Here we provide the derivation of the formula (25) for dP/dt. (PDF) [file pone.0207764.s001.pdf]

S1 Text

Here we provide the derivation of the formula (25) for dP/dt. Differentiation of (12) yields the following equation:

$$\frac{dF}{dt}\sum_{i=1}^{G}(1-k_i)N_i + F\left(-\sum_{i=1}^{G}\frac{dk_i}{dt}N_i + \sum_{i=1}^{G}(1-k_i)\frac{dN_i}{dt}\right)$$
$$= \sum_{i=1}^{G}\frac{dk_i}{dt}W_i + \sum_{i=1}^{G}k_i\frac{dW_i}{dt} - \frac{dP}{dt}\sum_{i=1}^{G}k_iN_i - P\sum_{i=1}^{G}\frac{dk_i}{dt}N_i - P\sum_{i=1}^{G}k_i\frac{dN_i}{dt}$$

Using the quasi-steady approximation, we may substitute $P + O(\tau)$ for F (i.e., (19)), $N_i\frac{dP}{dt} + O(\tau)$ for $\frac{dW_i}{dt}$ (i.e., (21)), and employ the constraint equation $\sum_{i=1}^{G}\frac{dN_i}{dt} = 0$ to obtain, respectively,

$$\frac{dP}{dt}\sum_{i=1}^{G}N_i + P\sum_{i=1}^{G}\frac{dN_i}{dt} = \sum_{i=1}^{G}\frac{dk_i}{dt}W_i + \sum_{i=1}^{G}k_i\frac{dW_i}{dt} + O(\tau)$$
$$\frac{dP}{dt}\sum_{i=1}^{G}N_i + P\sum_{i=1}^{G}\frac{dN_i}{dt} = \sum_{i=1}^{G}\frac{dk_i}{dt}W_i + \sum_{i=1}^{G}k_iN_i\frac{dP}{dt} + O(\tau)$$
$$\frac{dP}{dt}\sum_{i=1}^{G}(1-k_i)N_i = \sum_{i=1}^{G}\frac{dk_i}{dt}W_i + O(\tau)$$

The last form of the equality is solved for $\frac{dP}{dt}$, and with the substitution of $\frac{k_iW_i}{P}$ for N_i (i.e., (23)) one obtains (25).
