# Supplementary material for: Slow-fast analysis of a multi-group asset flow model with implications for the dynamics of wealth
Source: PLoS One. 2018 Nov 29;13(11):e0207764. doi: 10.1371/journal.pone.0207764 (PMC6264481; doi:10.1371/journal.pone.0207764)
Supplement: S2 Text — Here we present the statement and proof Lemma 1. (PDF) [file pone.0207764.s002.pdf]

S2 Text

Lemma 1. *Let $(P(t), \mathbf{W}(t))$ be a solution of the system (25)-(26) on the interval $[0, T]$ with trading rates $\mathbf{k}(t)$ and initial conditions $P(0) = P_0$, $\mathbf{W}(0) = \mathbf{W}_0$. It follows that*

(i) $d\mathbf{k}(t)/dt \cdot \mathbf{W}(t) > (<)\ 0$ *for* $t \in [0, T]$ *if and only if* $dP(t)/dt > (<)\ 0$ *and, consequently,* $dW_i(t)/dt > (<)\ 0$ *for* $t \in [0, T]$ *for all* i, $1 \le i \le G$.

(ii) *If* $(\mathbf{k}(t) - \mathbf{k}(0)) \cdot \mathbf{W}_0 = 0$ *for* $t \in [0, T]$, *then* $\mathbf{W}(t) = \mathbf{W}_0$ *for* $t \in [0, T]$.

(iii) *If* $dk_i(t)/dt = 0$ *for* $t \in [0, T]$ *and some* i, $1 \le i \le G$, *then*

$$\frac{W_i(t)}{W_{i,0}} = \left(\frac{P(t)}{P_0}\right)^{k_i}, \qquad \forall t \in [0, T]$$

In particular, if $P(T) = P_0$ *then* $W_i(T) = W_{i,0}$.

(iv) *If there are constants* $\alpha_1, \alpha_2, ..., \alpha_G, \beta$ *such that* $\sum_{j=1}^{G} \alpha_j k_j(t) = \beta$ *for* $t \in [0, T]$, *then*

$$\left(\frac{W_1(t)}{W_{1,0}}\right)^{\alpha_1} \left(\frac{W_2(t)}{W_{2,0}}\right)^{\alpha_2} \cdots \left(\frac{W_G(t)}{W_{G,0}}\right)^{\alpha_G} = \left(\frac{P(t)}{P_0}\right)^{\beta}, \qquad \forall t \in [0, T]$$

(v) *If there are nonzero constants* $\eta_1, \eta_2, ..., \eta_G$, *and a function* $f(t)$ *such that* $k_i(t) = k_{i,0} + \eta_i f(t)$ *for* $t \in [0, T]$, *then for any* i, $1 \le i \le G$,

$$\left(\frac{W_i(t)}{W_{i,0}}\right)^{\frac{1}{\eta_i}} \left(\frac{P(t)}{P_0}\right)^{-\frac{k_{i,0}}{\eta_i}} = g(P(t))$$

where $g(P)$ *is defined implicitly as the function of* P *that satisfies the following relation (for all* P*):*

$$\sum_{i=1}^{G} W_{i,0} \left(\frac{P}{P_0}\right)^{k_{i,0}} g(P)^{\eta_i} = \bar{M} + \bar{N} P.$$

(vi) *For any* i, j, $1 \le i, j \le G$, *the ratio* $W_i(t)/W_j(t)$ *instantaneously increases with* t *if and only if* $(k_i(t) - k_j(t)) \frac{dP}{dt} > 0$.

(vii) *If* $(\tilde{P}(t), \tilde{\mathbf{W}}(t))$ *is a solution of (25)-(26) with initial conditions* $P(0) = \beta P_0$, $\mathbf{W}(0) = \alpha \mathbf{W}_0$, *where* $\alpha, \beta > 0$, *and with trading rates* $\tilde{\mathbf{k}}(t) = \mathbf{k}(\sigma(t))$ *where* $\sigma(t)$ *is a monotone increasing differentiable function, then*

$$(\tilde{P}(t), \tilde{\mathbf{W}}(t)) = (\beta P(\sigma(t)), \alpha \mathbf{W}(\sigma(t)))$$

Proof. All of the propositions are self-evident. Proposition (i) follows from (25) and the positivity of $k_i W_i / P$. Proposition (ii) is verified by differentiation and comparison with (25)-(26). Proposition (iii) is obtained by integrating (26) with k_j assumed constant. Proposition (iv) follows from the combination of the hypothesis and (26) and subsequent integration. Proposition (v) follows from the combination of the hypothesis and (26), followed by integration, and the functional form of $g(P)$ is obtained using (27). Proposition (vi) is verified by differentiation and comparison with (26). Finally (vii) is verified by application of the chain rule to (25)-(26). □
