# Supplementary material for: Slow-fast analysis of a multi-group asset flow model with implications for the dynamics of wealth
Source: PLoS One. 2018 Nov 29;13(11):e0207764. doi: 10.1371/journal.pone.0207764 (PMC6264481; doi:10.1371/journal.pone.0207764)
Supplement: S3 Text — Here we present the statement and proof of Theorem 3. (PDF) [file pone.0207764.s003.pdf]

S3 Text

Theorem 3. *Let* $(P(t), \mathbf{W}(t))$ *be a solution of the system (25)-(26) on the interval* $[0, T]$ *with initial conditions* $P(0) = P_0$*,* $\mathbf{W}(0) = \mathbf{W}_0$ *and trading rates* $\mathbf{k}(t)$ *such that* $dk_i(t)/dt = 0$ *for* $t \in [0, T]$*,* $3 \leq i \leq G$*, while* $k_1(t)$ *and* $k_2(t)$ *on the interval* $[0, T]$ *form a closed curve* γ *in the* (k_1, k_2) *plane. Then for* $3 \leq i \leq G$*,*

$$\operatorname{sgn}(W_2(T) - W_{2,0}) = -\operatorname{sgn}(W_1(T) - W_{1,0}),$$
$$\operatorname{sgn}(W_i(T) - W_{i,0}) = \operatorname{sgn}(P(T) - P_0) = \operatorname{sgn}((W_1(T) - W_{1,0})(k_1(0) - k_2(0)))$$

Proof. In view of the definitions of $\hat{M}(P)$ and $\hat{N}(P)$ used in (37) and (38), the function $\hat{M}(P)$ is monotone non-increasing in P at fixed k_1, k_2, and $P\hat{N}(P)$ is monotone increasing in P at fixed k_1, k_2. Thus, $W_1(P)$, as given by the formula (37), is monotone increasing in P (at fixed k_1, k_2) as long as $k_1 > k_2$ and monotone decreasing in P (at fixed k_1, k_2) when $k_1 < k_2$. Suppose that $W_1(T) > W_{1,0}$ and $k_1(T) = k_1(0) > k_2(0) = k_2(T)$. Then, $W_1(P)$ is monotone increasing with P which implies that $P(T) > P_0$ and, by Lemma 1(iii), $W_i(T) > W_{i,0}$ for $2 < i \leq G$. If $W_1(T) > W_{1,0}$ and $k_1(0) < k_2(0)$, then W_1 is monotone decreasing with P and hence $P(T) < P_0$ and, by Lemma 1(iii), $W_i(T) < W_{i,0}$ for $2 < i \leq G$. The results for cases when $W_1(T) < W_{1,0}$ are obtained analogously. The monotonicity of $\hat{M}(P)$ and $P\hat{N}(P)$ and formula (38) also imply that $W_2(T) - W_{2,0}$ has the opposite sign to $W_1(T) - W_{1,0}$, no matter what the difference $k_1(0) - k_2(0)$ is. If $k_1(0) = k_2(0)$ then (39) implies $P(T) = P_0$ and, by Lemma 1(iii), $W_i(T) = W_{i,0}$. □
