# Supplementary material for: Slow-fast analysis of a multi-group asset flow model with implications for the dynamics of wealth
Source: PLoS One. 2018 Nov 29;13(11):e0207764. doi: 10.1371/journal.pone.0207764 (PMC6264481; doi:10.1371/journal.pone.0207764)
Supplement: S4 Text — Here we present the statement and proof of Lemma 4. (PDF) [file pone.0207764.s004.pdf]

S4 Text

Lemma 4. *Let $(P(t), \mathbf{W}(t))$ be a solution of the system (25)-(26) on the interval $[0, T]$ with initial conditions $P(0) = P_0$, $\mathbf{W}(0) = \mathbf{W}_0$ and trading rates $\mathbf{k}(t)$ such that $dk_i(t)/dt = 0$ for $t \in [0, T]$, $3 \le i \le G$, while $k_1(t)$ and $k_2(t)$ on the interval $[0, T]$ form a path in the $\mathbf{k}$-space that is a counterclockwise labeled quadrilateral ABCD with vertices $A = \mathbf{k}(0) = \mathbf{k}(T)$, $B = \mathbf{k}(t_B)$, $C = \mathbf{k}(t_C)$, $D = \mathbf{k}(t_D)$, where $0 < t_B < t_C < t_D < T$, the side AB is orthogonal to $\mathbf{W}_0$, the sides BC and DA lie on distinct lines passing through the origin, the side CD is orthogonal to $\mathbf{W}(t_C)$, and A is the vertex with the largest k_1. Then $W_1(T) > W_{1,0}$ and $W_2(T) < W_{2,0}$.*

Proof. By Lemma 1(ii) and (iv) (see (41))

$$W_2(t_C) = W_2(t_B)\left(\frac{W_1(t_C)}{W_1(t_B)}\right)^{\frac{k_2(t_B)}{k_1(t_B)}} = W_{2,0}\left(\frac{W_1(t_C)}{W_{1,0}}\right)^{\frac{k_2(t_B)}{k_1(t_B)}}$$

In addition, by Lemma 1(ii) and (iv),

$$W_2(T) = W_2(t_D)\left(\frac{W_1(T)}{W_1(t_D)}\right)^{\frac{k_2(t_D)}{k_1(t_D)}} = W_2(t_C)\left(\frac{W_1(T)}{W_1(t_C)}\right)^{\frac{k_2(t_D)}{k_1(t_D)}}$$

Note that since CD is orthogonal to $\mathbf{W}(t_C)$, it has a negative slope, and from the definition of the quadrilateral it follows that, $k_1(t_C) < k_1(t_D)$ and $k_2(t_C) > k_2(t_D)$, and hence

$$\frac{k_2(0)}{k_1(0)} = \frac{k_2(t_D)}{k_1(t_D)} < \frac{k_2(t_C)}{k_1(t_C)} = \frac{k_2(t_B)}{k_1(t_B)}$$

In addition, note that $d\mathbf{k}(t)/dt \cdot \mathbf{W}(t) < 0$ for $t_B < t < t_C$ which, in view of Lemma 1(i) implies that $W_1(t_C) < W_1(t_B)$. It follows that

$$\begin{aligned}
W_2(T) &= W_{2,0}\left(\frac{W_1(t_C)}{W_{1,0}}\right)^{\frac{k_2(t_B)}{k_1(t_B)}}\left(\frac{W_1(T)}{W_1(t_C)}\right)^{\frac{k_2(t_D)}{k_1(t_D)}} \\
&= W_{2,0}\frac{W_1(T)^{\frac{k_2(t_D)}{k_1(t_D)}}}{W_{1,0}^{\frac{k_2(t_B)}{k_1(t_B)}}}W_1(t_C)^{\frac{k_2(t_B)}{k_1(t_B)}-\frac{k_2(t_D)}{k_1(t_D)}} \\
&< W_{2,0}\frac{W_1(T)^{\frac{k_2(t_D)}{k_1(t_D)}}}{W_{1,0}^{\frac{k_2(t_B)}{k_1(t_B)}}}W_{1,0}^{\frac{k_2(t_B)}{k_1(t_B)}-\frac{k_2(t_D)}{k_1(t_D)}} \\
&= W_{2,0}\left(\frac{W_1(T)}{W_{1,0}}\right)^{\frac{k_2(t_D)}{k_1(t_D)}}
\end{aligned}$$

and hence we obtain the inequality

$$\frac{W_2(T)}{W_{2,0}} < \left(\frac{W_1(T)}{W_{1,0}}\right)^{\frac{k_2(0)}{k_1(0)}} \tag{42}$$

It follows from (37) that for a cyclic path, $W_1(T) - W_{1,0}$ has the opposite sign from $W_2(T) - W_{2,0}$, since

$$W_1(T) - W_{1,0} = \frac{\left[P(T)\hat{N}(P(T)) - P_0\hat{N}(P_0)\right](1 - k_2(0)) - \left[\hat{M}(P(T)) - \hat{M}(P_0)\right]k_2(0)}{k_1(0) - k_2(0)}$$

$$W_2(T) - W_{2,0} = \frac{\left[\hat{M}(P(T)) - \hat{M}(P_0)\right]k_1(0) - \left[P(T)\hat{N}(P(T)) - P_0\hat{N}(P_0)\right](1 - k_1(0))}{k_1(0) - k_2(0)}$$

and $P\hat{N}(P)$ is increasing in P while $\hat{M}(P)$ is non-increasing in P. Only the case when $W_1(T) > W_{1,0}$ and $W_2(T) < W_{2,0}$ is compatible with (42). □
