# Supplementary material for: Slow-fast analysis of a multi-group asset flow model with implications for the dynamics of wealth
Source: PLoS One. 2018 Nov 29;13(11):e0207764. doi: 10.1371/journal.pone.0207764 (PMC6264481; doi:10.1371/journal.pone.0207764)
Supplement: S5 Text — Here we present the statement and proof of Theorem 5, and the statement and proof of a lemma utilized in the proof of that theorem. (PDF) [file pone.0207764.s005.pdf]

S5 Text

Theorem 5. *Let $(P(t), \mathbf{W}(t))$ be a solution of the system (25)-(26) on the interval $[0, T]$ with initial conditions $P(0) = P_0$, $\mathbf{W}(0) = \mathbf{W}_0$ and trading rates $\mathbf{k}(t)$ such that $dk_i(t)/dt = 0$ for $t \in [0, T]$, $3 \leq i \leq G$, while $k_1(t)$ and $k_2(t)$ on the interval $[0, T]$ form a piecewise smooth Jordan curve γ in the (k_1, k_2) plane. Then $W_1(T) - W_{1,0} > 0$ (and consequently $W_2(T) - W_{2,0} < 0$) if and only if γ is traveled counterclockwise.*

Proof. We will begin by showing that the statement holds when the diameter of the path is sufficiently small, and then utilize Lemma 6 which is stated and proved below.

Consider first a piecewise smooth convex Jordan path γ of diameter $\epsilon > 0$ that encloses a portion of the (k_1, k_2) plane labeled Γ. Let $k_1(t)$ and $k_2(t)$ with $t \in [0, T]$ be a continuously differentiable parametrization of γ such that γ is traveled counterclockwise as t increases, and C be constant such that $|dk_i/dt| < C\epsilon$ and $|k_i(t) - k_{i,0}| < C\epsilon$, where $k_{i,0} = k_i(0)$. The integral form of (25)-(26),

$$W_i(T) - W_{i,0} = \int_0^T k_i(t) W_i(t) \frac{\sum_{j=1}^G W_j(t) \frac{dk_j(t)}{dt}}{\sum_{j=1}^G (1 - k_j(t)) k_j(t) W_j(t)} \, dt \tag{43}$$

leads to the estimate

$$|W_i(T) - W_{i,0}| \leq \int_0^T W_i(t) \frac{K_i C\epsilon}{(1-K)\kappa} \, dt \tag{44}$$

where $K_i = \max_{t \in [0,T]} k_i(t)$, $K = \max_{i=1,\ldots,G} K_i$, and $\kappa = \min_{i=1,\ldots,G} (\min_{t \in [0,T]} k_i(t))$. Application of Gronwall's inequality then provides bounds on the values attained by $W_i(t)$ on the interval $[0, T]$:

$$\exp\left(-\frac{K_i C\epsilon}{(1-K)\kappa} t\right) \leq \frac{W_i(t)}{W_{i,0}} \leq \exp\left(\frac{K_i C\epsilon}{(1-K)\kappa} t\right) \qquad t \in [0, T] \tag{45}$$

Consider now the case when $dk_i(t)/dt = 0$ for $t \in [0, T]$, $3 \leq i \leq G$. In view of (45) and the bounds on $|dk_i/dt|$ and $|k_i(t) - k_{i,0}|$, if we assume the following expansions

$$W_i(t) = \tilde{W}_i(k_1(t), k_2(t)) + O(\epsilon^2) \tag{46}$$

$$\tilde{W}_i(k_1, k_2) = W_{i,0} + a_{i1}(k_1 - k_{1,0}) + a_{i2}(k_2 - k_{2,0}) + O(\epsilon^2) \tag{47}$$

then (25)-(26) imply $a_{ij} = \frac{k_{i,0} W_{i,0} W_{j,0}}{\sum_{l=1}^G (1 - k_{l,0}) k_{l,0} W_{l,0}} > 0$. With these expansions we can rewrite (43) in a path-integral form and apply Green's lemma as follows

$$\begin{aligned} \Delta W_i = W_i(T) - W_{i,0} &= \oint_\gamma \frac{k_i \tilde{W}_i(k_1, k_2)}{S(k_1, k_2)} \left[\tilde{W}_1(k_1, k_2) dk_1 + \tilde{W}_2(k_1, k_2) dk_2\right] + O(\epsilon^4) \\ &= \iint_\Gamma \left(\frac{\partial}{\partial k_1} \frac{k_i \tilde{W}_i(k_1, k_2) \tilde{W}_2(k_1, k_2)}{S(k_1, k_2)} - \frac{\partial}{\partial k_2} \frac{k_i \tilde{W}_i(k_1, k_2) \tilde{W}_1(k_1, k_2)}{S(k_1, k_2)}\right) dk_1 dk_2 + O(\epsilon^4) \end{aligned} \tag{48}$$

where $S(k_1, k_2) = \sum_{j=1}^G (1 - k_j) k_j W_j(k_1, k_2)$. Evaluation of the integrand for $i = 1$

gives:

$$\Delta W_1 = \iint_\Gamma \left(\frac{\tilde{W}_1\tilde{W}_2 + k_1\tilde{W}_2 a_{11} + k_1\tilde{W}_1 a_{21}}{S} - \frac{k_1\tilde{W}_1\tilde{W}_2\left[(1-2k_1)\tilde{W}_1 + \sum_{j=1}^{G}(1-k_j)k_j a_{j1}\right]}{S^2} \right.$$

$$\left. - \frac{2k_1\tilde{W}_1 a_{12}}{S} + \frac{k_1\tilde{W}_1^2\left[(1-2k_2)\tilde{W}_2 + \sum_{j=1}^{G}(1-k_j)k_j a_{j2}\right]}{S^2} \right) dk_1 dk_2 + O(\epsilon^4)$$

$$= \frac{A(\Gamma)}{S_0^2}\left(W_{1,0}W_{2,0}S_0 + k_{1,0}W_{2,0}a_{11}S_0 + k_{1,0}W_{1,0}a_{21}S_0 - 2k_{1,0}W_{1,0}a_{12}S_0\right.$$

$$\left. - 2k_{1,0}W_{1,0}^2W_{2,0}\left[k_{2,0}-k_{1,0}\right] - k_{1,0}W_{1,0}\sum_{j=1}^{G}(1-k_j)k_j[a_{j1}W_{2,0} - a_{j2}W_{1,0}]\right) + O(\epsilon^3)$$

$$= \frac{A(\Gamma)W_{1,0}W_{2,0}}{S_0^2}\left((1-k_{2,0})k_{1,0}W_{1,0} + \sum_{j=2}^{G}(1-k_{j,0})k_{j,0}W_{j,0}\right) + O(\epsilon^3) \qquad (49)$$

where A is the area of domain Γ. Since the A is of order ϵ^2 and the lowest order term in (49) is always positive, the inequality $W_1(T) > W_{1,0}$ holds when ϵ is sufficiently small. Similarly, by evaluating the integrand in (48) for $i = 2$, one can show that within the lowest order in ϵ, $W_2(T) < W_{2,0}$.

Note also the important fact that the positivity of the integrand in (49), and hence the validity of $W_1(T) > W_{1,0}$, is independent of where on the curve γ we choose the starting point $t = 0$ and what the value of $W_{2,0}$ is.

Let us now define $V(\omega, V_0)$ as the ratio $W_1(T)/W_2(T)$ where $(P(t), \mathbf{W}(t))$ is a solution of the system (25)-(26) on the interval $[0, T]$ with initial conditions $P(0) = P_0$, $\mathbf{W}(0) = \mathbf{W}_0$, with $W_{1,0}/W_{2,0} = V_0$, and trading rates $\mathbf{k}(t)$ such that $dk_i(t)/dt = 0$ for $t \in [0, T]$, $3 \le i \le G$, while $k_1(t)$ and $k_2(t)$ on the interval $[0, T]$ form a piecewise smooth curve ω in the (k_1, k_2) plane. The function $V(\omega, V_0)$ is well defined since, by Lemma 1(vii), $\mathbf{W}(T)$ is independent of the parametrization of the curve ω and scaling invariant in $\mathbf{W}$. It is easy to verify that $V(\omega, V_0)$ satisfies the conditions of Lemma 6 (see below). We can therefore apply Lemma 6 (iii) and the above observations that $W_1(T) > W_{1,0}$ and $W_2(T) < W_{2,0}$, and hence $V(\omega, V_0) > V_0$ for any counterclockwise Jordan curve γ with sufficiently small diameter, to conclude that $W_1(T) > W_{1,0}$ for any counterclockwise Jordan curve γ for all $W_{1,0}$.

Suppose now that γ is oriented clockwise. Then the integral (49) is negative and hence $V(\omega, V_0) < V_0$ for any clockwise Jordan curve γ with sufficiently small diameter. Again, we use Lemma 6 (iii) to conclude that $W_1(T) > W_{1,0}$ for any clockwise Jordan curve γ for all $W_{1,0}$. $\square$

Statement and Proof of Lemma for Theorem 5

Lemma 6. *Let $\mathcal{S}$ be the space of oriented simple curves in $\mathbb{R}^2$. Consider a continuous map $V : \mathcal{S} \times \mathbb{R}_+ \to \mathbb{R}_+$ such that, for any $\omega \in \mathcal{S}$ and $V_0 \in \mathbb{R}_+$,*

$$V(-\omega, V(\omega, V_0)) = V_0 \tag{50}$$

(where $-\omega$ denotes ω traveled in the opposite direction) and for any disjoint subdivision $\omega_1 \cup \omega_2 = \omega$ (where ω_2 follows ω_1) and any $V_0 \in \mathbb{R}_+$,

$$V(\omega_1 \cup \omega_2, V_0) = V(\omega_2, V(\omega_1, V_0)) \tag{51}$$

Let γ be an oriented Jordan curve in $\mathbb{R}^2$. The following hold:

(i) The function $V(\omega, .) : \mathbb{R}_+ \to \mathbb{R}_+$ is one-to-one and monotone increasing for any fixed $\omega \in \mathcal{S}$.

(ii) If, for any $\omega \in S$ that covers γ, $\mathrm{sgn}(V(\omega, V_0) - V_0)$ is independent of V_0, then $\mathrm{sgn}(V(\omega, V_0) - V_0)$ is independent of ω for all $\omega \in S$ that cover γ with identical orientation.

(iii) If $\mathrm{sgn}(V(\omega, V_0) - V_0) = s$ for all $\omega \in S$ that cover counterclockwise oriented Jordan curves γ in $\mathbb{R}^2$ with diameter smaller than $\epsilon > 0$, then $\mathrm{sgn}(V(\omega, V_0) - V_0) = s$ for all $\omega \in S$ that cover any counterclockwise oriented Jordan curve in $\mathbb{R}^2$.

Proof. To prove (i) suppose that there exist V_0, $\bar{V}_0$, with $\bar{V}_0 > V_0$ such that $V(\omega, \bar{V}_0) < V(\omega, V_0)$. Then, by continuity of the map V, there is a disjoint subdivision $\omega_1 \cup \omega_2 = \omega$ such that $V(\omega_1, \bar{V}_0) = V(\omega_1, V_0)$. It follows from (50) that $\bar{V}_0 = V(-\omega_1, V(\omega_1, \bar{V}_0)) = V(-\omega_1, V(\omega_1, V_0)) = V_0$, which is a contradiction. Similarly, one obtains the one-to-one property.

To prove (ii), let ω be a curve covering γ and consider a disjoint subdivision $\omega_1 \cup \omega_2 = \omega$. Then $\bar{\omega} = \omega_2 \cup \omega_1$ also covers γ. Condition (51) implies that

$$\begin{aligned} V(\bar{\omega}, V(\omega_1, V_0)) &= V(\omega_1 \cup \bar{\omega}, V_0) \\ &= V(\omega \cup \omega_1, V_0) \\ &= V(\omega_1, V(\omega, V_0)) \end{aligned}$$

Now, by (i), $\mathrm{sgn}(V(\omega_1, V(\omega, V_0)) - V(\omega_1, V_0)) = \mathrm{sgn}(V(\omega, V_0) - V_0)$. Therefore, $\mathrm{sgn}(V(\bar{\omega}, V(\omega_1, V_0)) - V(\omega_1, V_0)) = \mathrm{sgn}(V(\omega, V_0) - V_0)$. Consequently, if $V(\omega, V_0) - V_0$ has the same sign for all V_0, then, by virtue of $V(\bar{\omega}, .)$ being one-to-one, same is true for $V(\bar{\omega}, V_0) - V_0$.

Now, to prove (iii), consider a counterclockwise oriented Jordan curve ω in (k_1, k_2) plane. Let A and B be distinct points on ω, let ω_1 be the oriented segment of ω from A to B, ω_2 be the oriented segment of ω with from B back to A, and ω_3 be a simple, oriented, non-intersecting curve from A to B that lies in the interior Ω of ω. Then ω_3 divides Ω into two disjoint regions Ω_1 and Ω_2 which have boundaries $\partial\Omega_1 = \omega_1 \cup (-\omega_3)$ and $\partial\Omega_2 = \omega_3 \cup \omega_2$, respectively, both counterclockwise oriented Jordan curves. Conditions (50) and (51) imply

$$\begin{aligned} V(\omega_1 \cup \omega_2, V_0) &= V(\omega_2, V(\omega_1, V_0)) \\ &= V(\omega_2, V(\omega_3, V(-\omega_3, V(\omega_1, V_0)))) \\ &= V(\omega_3 \cup \omega_2, V(\omega_1 \cup (-\omega_3), V_0)) \end{aligned}$$

It follows that if $\text{sgn}(V(\omega_1 \cup (-\omega_3), V_0) - V_0) = \text{sgn}(V(\omega_3 \cup \omega_2, V_0) - V_0) = s$ for all V_0, then $\text{sgn}(V(\omega_1 \cup \omega_2, V_0) - V_0)$ will be independent of V_0 (and identical to s). Using (ii) we can conclude that $\text{sgn}(V(\omega_1 \cup \omega_2, V_0) - V_0) = s$ for any choice of the points A and B. Consider now a decomposition of Ω into disjoint regions Ω_i, $i = 1, ..., Q$ such that the boundaries $\partial\Omega_i$ are equally oriented Jordan curves of diameter smaller than ϵ, and such that, for each $i = 1, ..., Q$, $\text{sgn}(V(\omega; V_0) - V_0) = s$ for all V_0 and all $\omega \in \mathcal{S}$ that cover $\partial\Omega_i$. Since Ω is a simply connected region, by induction over the compositions of neighboring subdomains Ω_i we can conclude that $\text{sgn}(V(\omega; V_0) - V_0) = s$ for all V_0. $\square$
