# Supplementary material for: Slow-fast analysis of a multi-group asset flow model with implications for the dynamics of wealth
Source: PLoS One. 2018 Nov 29;13(11):e0207764. doi: 10.1371/journal.pone.0207764 (PMC6264481; doi:10.1371/journal.pone.0207764)
Supplement: S6 Text — Here we present the statement and proof of Theorem 6. (PDF) [file pone.0207764.s006.pdf]

S6 Text

Theorem 6. *Let* $(P(t), \mathbf{W}(t))$ *be a solution of the system (25)-(26) on the interval* $[0, T]$ *with initial conditions* $P(0) = P_0$*,* $\mathbf{W}(0) = \mathbf{W}_0$ *and trading rates* $\mathbf{k}(t)$ *such that* $dk_i(t)/dt = 0$ *for* $t \in [0, T]$*,* $3 \leq i \leq G$*. For any piecewise smooth non-constant cyclic strategy* $k_2(t)$*,* $t \in [0, T]$*, and any* K *there exists a cyclic strategy* $k_1(t)$ *with* $k_1(0) = k_1(T) = K$ *such that* $W_1(T) - W_{1,0} > 0$ *and* $W_2(T) - W_{2,0} < 0$*.*

Proof. Without loss of generality assume that $k_2(t)$ attains its global minimum on $[0, T)$ at the value $t = 0$ and that this minimum is isolated and unique. Set

$$k_1(t) = \begin{cases} K\left(1 + \frac{t}{\epsilon}\right) & 0 \leq t < \epsilon \\ 2K\left(1 - \frac{t-\epsilon}{T-2\epsilon}\right) & \epsilon \leq t < T - \epsilon \\ K\left(1 + \frac{t-T}{\epsilon}\right) & T - \epsilon \leq t \leq T \end{cases}$$

Note that $k_1(\epsilon)$ is the unique isolated global maximum of $k_1(t)$, that $k_1(T - \epsilon)$ is the unique global minimum of $k_1(t)$, and that $k_1(t)$ is monotone increasing on $[0, \epsilon]$ and $[T - \epsilon, T]$ and monotone decreasing on $[\epsilon, T - \epsilon]$. The functions $k_1(t)$ and $k_2(t)$ on the interval $[0, T]$ form a piecewise smooth curve γ in the (k_1, k_2) plane. By continuity and smoothness of $k_2(t)$, for sufficiently small $\epsilon > 0$, all interior local minima of $k_2(t)$ in the interval $(\epsilon, T - \epsilon)$ are larger than both $k_2(\epsilon)$ and $k_2(T - \epsilon)$ (since $\max\{k_2(\epsilon), k_2(T - \epsilon)\} \to k_2(0)$ as $\epsilon \to 0$ and 0 is not an accumulation point of local minima of $k_2(t)$). It follows that the segment γ_1 of γ with $t \in (T - \epsilon, T] \cup [0, \epsilon)$ does not intersect the segment γ_2 of γ with $t \in (\epsilon, T - \epsilon)$, and, by the monotonicity properties of $k_1(t)$, γ_1 and γ_2 do not self-intersect. It follows that γ is a Jordan curve that is traveled counterclockwise and Theorem 5 implies the desired result.

If the minimum is not unique, then $[0, T]$ can be divided into subintervals on which the minima are unique. For each such subinterval one can construct the corresponding $k_1(t)$ and complete the proof as above. If the minimum is not isolated then ϵ must be large enough to contain the interval on which $k_1(t)$ is constant. □
